# The tumor–stroma ratio and the immune microenvironment improve the prognostic prediction of pancreatic ductal adenocarcinoma

**DOI:** 10.1007/s12672-023-00744-w

**Published:** 2023-07-05

**Authors:** Mei Lu, Yi Zou, Peiling Fu, Yuyang Li, Pengcheng Wang, Guoping Li, Sheng Luo, Yupeng Chen, Guoping Guan, Sheng Zhang, Linying Chen

**Affiliations:** 1 Department of Pathology, the First Affiliated Hospital of Fujian Medical University, Fuzhou, 350005 Fujian China; 2 Fuqing City Hospital Affiliated to Fujian Medical University, Fuqing, Fujian China; 3 Department of Pathology, Second Affiliated Hospital Zhejiang University School of Medicine, Hangzhou, Zhejiang China; 4 Department of Pathology, National Regional Medical Center, Binhai Campus of the First Affiliated Hospital of Fujian Medical University, Fuzhou, Fujian China; 5 Fujian Key Laboratory of Translational Research in Cancer and Nurodegernerative Diseases, Fuzhou, Fujian China

**Keywords:** Pancreatic ductal adenocarcinoma, Tumor–node–metastasis staging, Tumor–stroma ratio, Tumor microenvironment, Prognosis

## Abstract

Tumor-infiltrating immune cells and fibroblasts are significant components of the tumor microenvironment (TME) of pancreatic ductal adenocarcinoma (PDAC), and they participate in tumor progression as closely as tumor cells. However, the relationship between the features of the TME and patient outcomes and the interactions among TME components are still unclear. In this study, we evaluated the PDAC TME in terms of the quantity and location of cluster of differentiation (CD)4^+^ T cells, CD8^+^ T cells, macrophages, stromal maturity, and tumor-stroma ratio (TSR), as evaluated by immunohistochemical staining of serial whole-tissue sections from 116 patients with PDAC. The density of T cells and macrophages (mainly activated macrophages) was significantly higher at the invasive margins (IMs) than at the tumor center (TC). CD4^+^ T cells were significantly association with all the other tumor-associated immune cells (TAIs) including CD8, CD68 and CD206 positive cells. Tumors of the non-mature (intermediate and immature) stroma type harbored significantly more CD8^+^ T cells at the IMs and more CD68^+^ macrophages at the IMs and the TC. The density of CD4^+^, CD8^+^, and CD206^+^ cells at the TC; CD206^+^ cells at the IMs; and tumor-node-metastasis (TNM) staging were independent risk factors for patient outcomes, and the c-index of the risk nomogram for predicting the survival probability based on the TME features and TNM staging was 0.772 (95% confidence interval: 0.713–0.832). PDAC harbored a significantly immunosuppressive TME, of which the IMs were the hot zones for TAIs, while cells at the TC were more predictive of prognosis. Our results indicated that the model based on the features of the TME and TNM staging could predict patient outcomes.

**Supplementary Information:**

The online version contains supplementary material available at 10.1007/s12672-023-00744-w.

## Introduction

Pancreatic ductal adenocarcinoma (PDAC) is a devastating disease that ranks as the fourth leading cause of cancer-associated mortality in the United States [1]. Despite great improvements in the diagnosis and treatment for PDAC, the clinical outcomes of patients with this disease remain poor, even for patients treated at an early stage. Although extensive efforts have been made to identify molecular markers to improve the risk prediction of patients with PDAC, their clinical use needs to be further investigated. The traditional tumor–node–metastasis (TNM) staging system is widely used to guide the clinical treatment and evaluate the prognosis of patients with PDAC. The TNM staging system was originally proposed by Pierre Denoix in the mid-twentieth century [2], and it is periodically updated by the Union for International Cancer Control and the American Joint Committee on Cancer [3]. According to the tumor burden, including the tumor size, number of involved lymph nodes, degree of invasion, and distant metastatic spread, patients are grouped into four clinical stages, each of which has its own suggested management strategies after surgery. However, the TNM staging system does not provide complete prognostic information for the existing variable clinical outcomes in patients, even patients with the same TNM stage and similar treatment regimens [4, 5]. Nowadays, it is suspected that the components of the tumor microenvironment (TME) could effectively predict prognosis in patients with PDAC.

The TME of PDAC is characterized by prominent dense fibrotic desmoplasia, cancer-associated fibroblasts (CAFs), extensive extracellular matrix (ECM), and various infiltrating immune cells, which account for the majority of the tumor volume. As important orchestrators in the TME, CAFs exert their effects on the tumor structure to remodel the TME. Specifically, CAFs secrete ECM proteins and chemicals that contribute to tumor progression [6–8]. The CAF population exhibits great heterogeneity; thus, the roles of these cells in the development of PDAC remain under debate [9, 10]. Although more delicate molecular subgroups of CAFs, including myofibroblast-type, inflammatory-type, and antigen-presenting-type, as well as their functions in PDAC, have been explored, it is still a certain distant way in clinical application due to the lack of robust and effective biomarkers [11, 12]. Recent studies have shown that tumor stromal maturity and the tumor–stroma ratio (TSR) reflect the heterogeneous features of the stroma, and they have been indicated as prognostic factors and immunomodulators, respectively, for PDAC [13]. Due to their simple and economic assessment with conventional hematoxylin and eosin (H&E) staining, these methods are convenient possibilities for future clinical application. However, the TME is complex and highly heterogeneous. With regard to the impact on clinical outcomes in patients with PDAC, tumor-associated immune cells (TAIs) within the stroma need to be fully considered beyond the stromal features. Furthermore, their association with the density and location of TAIs should be clarified in patients undergoing immunotherapy. With regard to TAIs within the TME, T lymphocytes and tumor-associated macrophages (TAMs) are two essential cell populations. Evidence suggests that T lymphocytes within the TME influence the macrophage phenotype, and that macrophages accumulate or facilitate the spatial redistribution of cytotoxic T lymphocytes in pancreatic cancer [14, 15]. T lymphocytes are scattered in the tumor center (TC) within the stroma, in invasive margins (IMs), or in the organized lymphoid follicles distant from the tumor; thus, they demonstrate a distinctive spatial distribution. Previous studies have utilized spatial information to improve our understanding of TAM heterogeneity and highlight its correlation with patient outcomes to stratify patients with PDAC for effective immunotherapy [16, 17]. The immune score calculated based on the number of T lymphocytes and T lymphocyte subsets according to their location and density, as well as the striking spatial heterogeneity of TAMs, has influenced the survival of patients with colorectal cancer, gastric cancer, and melanoma [18–20]. Although some studies have demonstrated that the number of TAIs and different TAI subsets are associated with the prognosis of pancreatic cancer [21–23], studies on their spatial distribution in PDAC are limited. TAI heterogeneity is driven by different stimuli from TME cell subpopulations [8], which warrants an understanding of the roles of these TME cell subpopulations in anti-tumor immunity. However, their heterogeneous spatial distributions, interrelationships, and influences on the prognosis of PDAC remain uncertain.

To better understand the prognostic value of distinct TME features, including the degree of stromal maturity, the TSR, TAI populations, infiltrating T cell subsets, and TAMs in PDAC, whole tissue sections of surgically resected pancreatic cancer tissues were stained with H&E and subjected to a correlative immunohistochemistry analysis. We identified the degree of CAF maturity and the TSR to understand the stromal features. We also used CD4 and CD8 staining to mark T lymphocyte subsets, as well as CD68 and CD206 staining to mark pan-macrophages and M2-like macrophages, respectively. The locations of infiltrating immune cells in the TC and IMs were explored, and their relationship with clinical outcomes in patients with PDAC were observed. Furthermore, the interrelationships between the stromal features and the spatial distribution and subsets of infiltrating immune cells were also analyzed.

## Materials and methods

### Study population

One hundred sixteen consecutive patients diagnosed with PDAC who underwent surgical excision at the First Affiliated Hospital of Fujian Medical University from 2006 to 2016 were included. All of the patients did not undergo neoadjuvant therapy before surgery. For the tumor locations, 95 cases were in the head of pancreas and 21 were in the body or tail. Of them, 92 patients received whipple operation and 24 patients received pancreatectomy or tumor resections. For surgical pathology, PDAC tumor specimens were embedded if the tumor diameter was less than 20 mm. One representative section was embedded for every 10 mm if the diameter was more than 20 mm. Each formalin-fixed paraffin-embedded block was cut into 4-μm-thick slices and stained with H&E. All original H&E slides were reviewed, and the representative block was selected, which included the tumor and the adjacent healthy tissues. The histological diagnoses and grades were confirmed according to the latest World Health Organization criteria [24]. Furthermore, lymph node involvement, nerve invasion, and extra-pancreatic invasion were evaluated. Patients’ demographic and clinical details, including age, pN stage, pT stage, and outcomes, were retrieved from their medical records. According to the threshold value of serum biomarkers serum CA199 ≤ 37U/L was defined as low whereas > 37U/L was high. Serum CEA ≤ 5 ng/ml was defined as Low, and > 5 ng/ml was high. A toal of eight patients had tumor metastasis when underwent operation. Among them, 4 patients had liver metastasis, 2 had retroperitoneal lymphatic metastasis, 1 had lung metastasis and 1 had duodenal metastasis (Table S1).Overall survival (OS) was defined as the interval from the date of the initial diagnosis to the date of death from PDAC. Median survival (MS) was referred to the time corresponding to a survival rate of 50 percentage. Patients who died within 1 month after surgery were excluded (Table S2).

### Evaluation of stromal maturity and TSR in PDAC

The stromal features, including stromal maturity and TSR, of tumor specimens stained with H&E were assessed by two pathologists (M. L. and L. Y. C.), who were blinded to the clinicopathological results. In case of disagreement, the final interpretation was reached by consensus using the multi-head microscope. The degree of stromal maturity was graded into three groups according to the proportion of activated CAFs, which are large, plump, spindle-shaped cells with prominent nucleoli on histopathology. The immature phenotype was defined when the proportion of activated fibroblasts was more than 50% of all fibroblasts. The stroma was considered as mature when it was mainly composed of dense powdered collagen matrix and the proportion of activated fibroblasts was less than 10%. The intermediate phenotype was defined as described previously [7]. For the TSR (tumor-stroma ratio) assessment, two observers (ML and LYC) independently scored the slides using a conventional light microscope according to a previously published protocol for TSR assessment [13]. Firstly, the whole tissue slide was examined and the five region of interest (ROI) with most representative invasive tumor areas containing high intratumoral stroma was selected. Subsequently, within ROI, the percentage of tumor epitheliums and the percentage of intratumor stroma were estimated in a semiquantitative manner at × 100 magnification (/3.80 mm^2^) and recorded in 10% increments. The average percentage was calculated and any inconsistent results were resolved by consensus. Meanwhile, in the process of TSR score, mucus, necrosis, larger vessels and normal pancreatic tissues in the ROI were excluded. A high stromal volume was considered when the ratio of the percentage of tumor epitheliums and the percentage of intratumor stroma was less than 1, whereas a low stromal volume was considered when the ratio was greater than 1 [13].

### Evaluation of TAI populations in PDAC

Two pathologists (M. L. and L. Y. C.) evaluated the TAI populations in PDAC following the recommendations of the International TILs Working Group 2014 [25]. Leukocytes (morphonuclear and polymorphonuclear leukocytes, including lymphocytes and plasma cells) were scored at 200 × magnification (/0.95 mm^2^) by microscopy. The total accumulation of stromal TAIs was scored as a percentage (%) between the areas of carcinoma on at least five hot-spot fields, and the average value was calculated. Peritumoral follicular aggregates and tertiary lymphoid structures with germinal centers were not included. Areas with necrosis and technical artifacts were also avoided.

### Immunohistochemical staining

One representative 4-μm-thick slide including the tumor and adjacent healthy pancreatic tissue was selected from each patient by two pathologists (M. L. and L. Y. C.). All of the slides were stained for CD4 (CD4/4B12, 1:100, Dako), CD8 (C8/144B, 1:100, Dako), CD68 (KP1, 1:100, Dako), and CD206 (ab64693, 1:2000, Abcam). Whole slides were prepared for immunohistochemistry according to the product protocol using monoclonal antibodies, as follows. After de-waxing, hydration, and antigen retrieval (EDTA at pH 8.0, 37 ℃ for 30 min), the immunohistochemistry protocol was performed using a Dako EnVision FLEX + detection system (DK-2600, Dako, Denmark) and an Ultraview Universal DAB Detection Kit (Ventana, AZ, US). All of the slides were re-stained with hematoxylin. Tonsil tissue was used as the positive control, and phosphate-buffered saline (instead of primary antibody) was used as the negative control.

### Quantification of immune cells

For quantification, the whole-tissue section containing both healthy pancreatic tissue and tumor tissue was included to evaluate immune cells. As previously described, the immune cell score was determined first. The IMs were defined as the area covered by 200 microscopic magnifications (BX53 Olympus microscope system) covering tumor and healthy pancreatic tissue when putting the border of the tumor at the center of the visual field. The TC was defined as the rest of the tumor tissue [26]. The whole tumor was defined as containing the IMs and the TC. To evaluate the quantity of stained immune cells, at least three high-power fields (200 × magnification for CD4 and CD8, 400 × magnification for CD68 and CD206) at the IMs and the TC with the maximum density were photographed. Immunohistochemical staining was assessed with blinding to the clinical data. Complete and clear membranous and/or cytoplasmic staining of a moderate to strong density was considered positive for all markers (CD4, CD8, CD68, and CD206). The positive cells located at the tertiary lymphoid structures were excluded, and areas with tumor necrosis and mucus extravasation were avoided. For membrane staining, the number of CD4^+^ and CD8^+^ T cells was counted using Qupath software (version 0.2.1) [27], whereas the number of CD68^+^ and CD206^+^ cells was counted manually for their cytoplasmic staining. The average number of positive immune cells per high-power field was calculated and converted to the cell density value (cells/mm^2^). After quantification of immune cells, the each group of immune cells was subjected to dichotomize into high and low categories. To achieve the optimal cut-off value for immune cells classification, “surv_cutpoint” of R “survminer”was used and the optimal cut-off number for each immune cell group was calculated based on the overall survival, respectively [28].

### Uniform manifold approximation and projection (UMAP) analysis

For UMAP, we used the R software package “UMAP” (version 0.2.7.0) for analysis. We first calculated the z-score on the expression spectrum, and we then used the UMAP function for dimension reduction analysis to obtain the reduced matrix.

### Statistical analysis

All statistical analyses were performed using R software (version 4.0.2). Differences in clinicopathological variables between groups were identified using the chi-square test or Fisher’s exact test, as appropriate. Spearman’s correlation was used to analyze the relationship between the stromal features and tumor-infiltrating immune cells. The survival analysis was performed using the Kaplan–Meier method combined with the log-rank test. Independent risk factors affecting OS were identified using the univariate and multivariate Cox regression analyses. The risk nomogram was used to predict the 1-year and 3-year OS of patients with PDAC. The Harrell concordance index (c-index) and calibration curves were generated to evaluate the performance of the nomogram. The receiver operating characteristic (ROC) curve was used to evaluate the discrimination of the nomogram. A *p* value of < 0.05 was considered statistically significant.

## Results

**Fig. 1:**
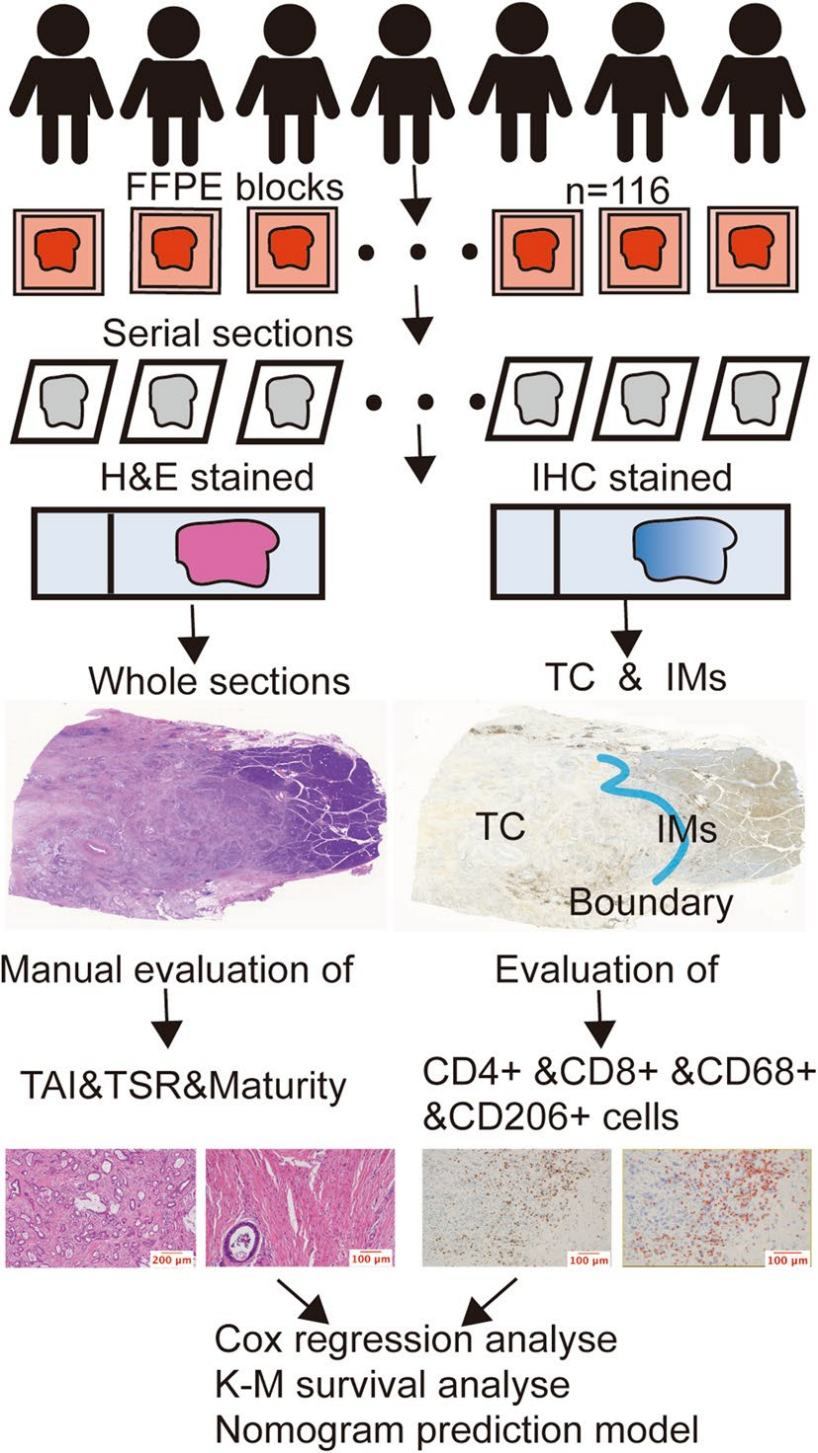
Study flowchart. One hundred sixteen patients were enrolled, and serial sections were stained by H&E and immunohistochemistry. The tumor center and invasive margins of the whole sections were defined and analyzed

To investigate the roles of stromal heterogeneity and immune-spatial distribution in PDAC, we evaluated the degree of stromal maturity, the TSR, the TAI populations, the infiltrating T cell subsets, and TAMs in PDAC. Whole-tissue sections of surgically resected pancreatic cancer specimens were used to evaluate H&E staining. The flowchart of this study is shown in Fig. 1. A total of 116 patients with PDAC were enrolled, including 70 males and 46 females. The median age of the patients was 60 years (range, 28–86 years), and the median tumor size was 3.3 cm (range, 0.7–11.0 cm). In terms of the histological grade, 30, 24, and 62 patients were graded well, moderate, and poor, respectively. In terms of the TNM stage, 19, 65, and 32 patients were classified as pT1, pT2, and pT3, respectively; 55, 48, and 13 patients were classified as pN0, pN1, and pN2, respectively; and 86 and 30 patients were classified as TNM stage I–II and III–IV, respectively. Follow-up data were available for 107 patients, among which 77 died of PDAC. The median OS time in total was 12.0 months (range, 1–84 months) (Tables S3–S6).

### Heterogeneous characteristics of stromal compartments in PDAC

**Fig. 2:**
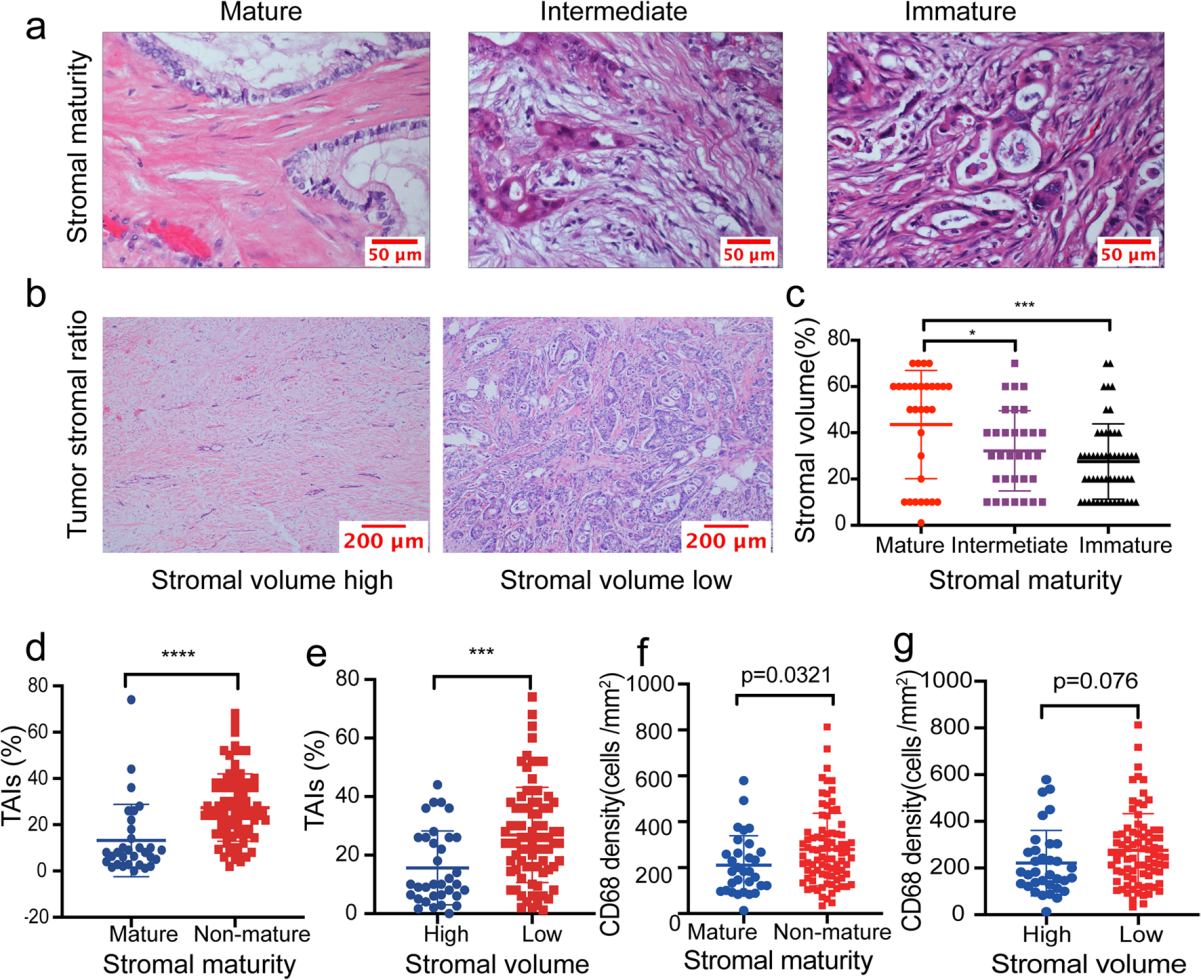
Phenotype of stromal volume and maturity, and their relationships with infiltrating immune cells in pancreatic ductal adenocarcinoma. **a** Representative pictures of tumor stromal subtypes: mature, intermediate, or immature (scale bar = 50 μm). **b** Representative pictures of the tumor–stroma ratio: high or low stromal volume (scale bar = 200 μm). **c** The relationships between stromal maturity and stromal volume, **d** stromal maturity and tumor-associated immune cells (TAIs), **e** stromal volume and TAIs, **f** stromal maturity and CD68^+^ cells in whole slides, **g** stromal volume and CD68^+^ cells in whole slides. Mann–Whitney U test, *p < 0.05, **p < 0.01, ***p < 0.001, ****p < 0.0001

PDAC is a type of solid tumor with an obvious desmoplastic stroma, which makes up the majority of the tumor size [29]. Microscopically, the regional TME exhibits marked heterogeneity with different degrees of ECM and various cellular components and morphological characteristics. In terms of the dominant stromal compartments in the TME, CAFs are trapped in eosinophilic abundant collagenous stroma or basophilic cellular collagen, which contains poor matrix. According to the percentage of activated CAFs (representative illustrations are shown in Fig. 2a), 31, 32, and 53 patients were defined as having mature, intermediate, and immature stromal phenotypes, respectively. For the TSR in PDAC, with the exception of one patient in whom the TSR was almost completely composed of tumor cells, the ratio ranged from 9:1 to 3:7. When the patients were grouped according to the TSR, 82 patients were classed as having a low stromal volume, whereas 34 were classed as having a high stromal volume (representative illustrations are depicted in Fig. 2b). The stromal maturity was significantly associated with the TSR in PDAC (Fig. 2c). Cases with an immature stromal phenotype exhibited a lower stromal volume. Stromal compartments play important roles in modulating and recruiting immune cells in PDAC [30]; thus, we explored the relationship between the stromal features and the immune composition of the tumor using whole-tissue H&E-stained and immunohistochemically stained sections. The results showed that a non-mature stromal type and a low stromal volume were significantly positively associated with TAIs infiltrating within the stroma in PDAC (both *p* < 0.0001) (Fig. 2d, e), particularly with an increase in CD68^+^ macrophage infiltration in the whole tumor (*p* = 0.0321 for stromal maturity [(Fig. 2f) and *p* = 0.076 for stromal volume (Fig. 2g)]. However, no significant results between the features of stroma and infiltrating immune cells were found for CD4^+^, CD8^+^, and CD206^+^ cells in the whole tumor (Figure S1).

### Distinct stromal features are associated with the spatiotemporal distribution of immune cells in PDAC

Previous studies have indicated that infiltrating immune cells are mediated by the stromal compartments in PDAC [7, 8]. We found that stromal features, including stromal maturity and volume, were closely related to CD68^+^ macrophage infiltration in the whole tumor area. However, whether the distinct stromal architecture influences the spatiotemporal distribution of immune cells remains unclear. For this purpose, we analyzed the number of tumor-infiltrating immune cells at the TC and IMs.

**Fig. 3:**
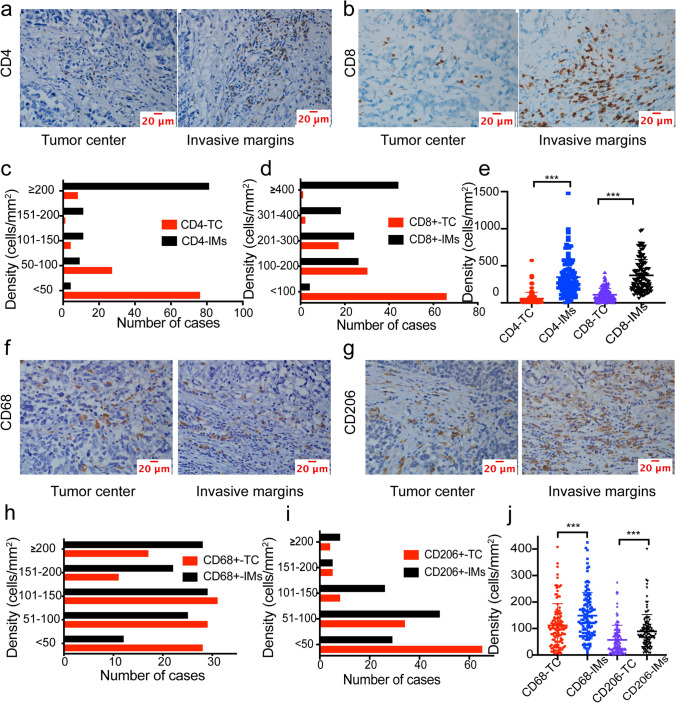
Distinct spatiotemporal distribution of immune cells in pancreatic ductal adenocarcinoma. **a** Representative pictures of CD4^+^ at the tumor center (TC) and invasive margins (IMs) (scale bar = 20 μm). **b** Representative pictures of CD8^+^ at the TC and IMs (scale bar = 20 μm). **c** Distributions of CD4^+^ T cells within the TC and IMs. **d** Distribution of CD8^+^ T cells at the TC and IMs. **e** Comparison of CD4^+^ and CD8^+^ cells at the TC and IMs, respectively. **f** Representative pictures of CD68^+^ at the TC and IMs (scale bar = 20 μm). **g** Representative pictures of CD206^+^ cells at the TC and IMs (scale bar = 20 μm). **h** Distribution of CD68^+^ cells within the TC and IMs. **i** Distribution of CD206^+^ cells at the TC and IMs. **j** Comparison of CD68^+^ and CD206^+^ cells at the TC and IMs, respectively. Mann–Whitney U test, *p < 0.05, **p < 0.01, ***p < 0.001, ****p < 0.0001

As illustrated by immunohistochemistry, CD4^+^, CD8^+^, CD68^+^, and CD206^+^ cells were detectable in stromal compartments with an abnormal distribution in all 116 patients (Fig. 3a, b, f, g; Figure S2, S3). For CD4^+^ T cells, the median number of cells was 32.00 cells/mm^2^ (range, 4–572 cells/mm^2^) at the TC and 284.00 cells/mm^2^ (range, 4–1477 cells/mm^2^) at the IMs. For CD8^+^ T cells, the median number was 87.00 cells/mm^2^ (range, 2–411 cells/mm^2^) at the TC and 318.00 cells/mm^2^ (range, 59–980 cells/mm^2^) at the IMs. The distribution of CD4^+^ and CD8^+^ T cells at the IMs and TC showed an opposite trend (Fig. 3c, d). The quantity of CD4^+^ and CD8^+^ T cells at the TC was significantly lower than at the IMs (*p* < 0.0001 for both) (Fig. 3e). For CD68^+^ macrophages, the median number was 100.00 cells/mm^2^ (range, 5–409 cells/mm^2^) at the TC and 134.00 cells/mm^2^ (range, 9–425 cells/mm^2^) at the IMs. For CD206^+^ macrophages, the median number was 38.00 cells/mm^2^ (range, 5–275 cells/mm^2^) at the TC and 75.00 cells/mm^2^ (range, 9–400 cells/mm^2^) at the IMs. The number of CD68^+^ and CD206^+^ macrophages at the IMs was significantly higher than at the TC (*p* = 0.0011 for CD68^+^ and *p* < 0.0001 for CD206^+^) (Fig. 3j). Tumor-infiltrating immune cells at the TC and IMs showed close associations with each other (all *p* < 0.05). Furthermore, CD4^+^ T cells were significantly positively correlated with CD8^+^, CD68^+^, and CD206^+^ infiltrating cells. Specifically, significant positive relationships between CD4_TC_^+^ T cells and CD8_TC_^+^ (RS = 0.317, *p* < 0.001), CD68_TC_^+^ (RS = 0.307, *p* < 0.001), CD206_TC_^+^ (RS = 0.278, *p* = 0.003), and CD206_IM_^+^ (RS = 0.271, *p* = 0.003) T cells were found. Accordingly, CD4_IM_^+^ T cells were closely positively correlated with CD8_IM_^+^ (RS = 0.277, *p* = 0.003), CD68_TC_^+^ (RS = 0.349, *p* < 0.001), CD68_IM_^+^ (RS = 0.227, *p* = 0.014), CD206_TC_^+^ (RS = 0.257, *p* = 0.005), and CD206_IM_^+^ (RS = 0.262, *p* = 0.005) cells (Table 1).

**Table 1:** Interrelationships between the density of the whole tumor and different regions of infiltrating immune cells in pancreatic ductal adenocarcinoma

RS	CD4			CD8			CD68			CD206		
p value	WT	TC	IMs	WT	TC	IMs	WT	TC	IMs	WT	TC	IMs
CD4:WT	NS	0.654	0.988	0.282	0.086	0.284	0.308	0.368	0.225	0.299	0.27	0.279
TC	< 0.001	NS	0.551	0.263	0.317	0.166	0.26	0.307	0.157	0.296	0.278	0.271
IMs	< 0.001	< 0.001	NS	0.26	0.04	0.277	0.297	0.349	0.227	0.285	0.257	0.262
CD8:WT	0.002	0.004	0.005	NS	0.592	0.945	0.36	0.366	0.256	0.26	0.242	0.245
TC	0.357	0.001	0.668	< 0.001	NS	0.322	0.192	0.226	0.096	0.204	0.222	0.168
IMs	0.002	0.074	0.003	< 0.001	< 0.001	NS	0.373	0.361	0.289	0.242	0.205	0.237
CD68:WT	0.001	0.005	0.001	< 0.001	0.039	< 0.001	NS	0.856	0.899	0.445	0.42	0.428
TC	< 0.001	0.001	< 0.001	< 0.001	0.015	< 0.001	< 0.001	NS	0.561	0.413	0.491	0.333
IMs	0.015	0.093	0.014	0.005	0.304	0.002	< 0.001	< 0.001	NS	0.389	0.276	0.434
CD206:WT	0.001	0.001	0.002	0.005	0.028	0.009	< 0.001	< 0.001	< 0.001	NS	0.856	0.886
TC	0.003	0.003	0.005	0.009	0.017	0.027	< 0.001	< 0.001	0.003	< 0.001	NS	0.549
IMs	0.002	0.003	0.005	0.008	0.071	0.01	< 0.001	< 0.001	< 0.001	< 0.001	< 0.001	NS

Spearman’s correlation was used for the analysis. *WT* whole tumor, *TC* tumor center, *IMs* invasive margins

**Fig. 4:**
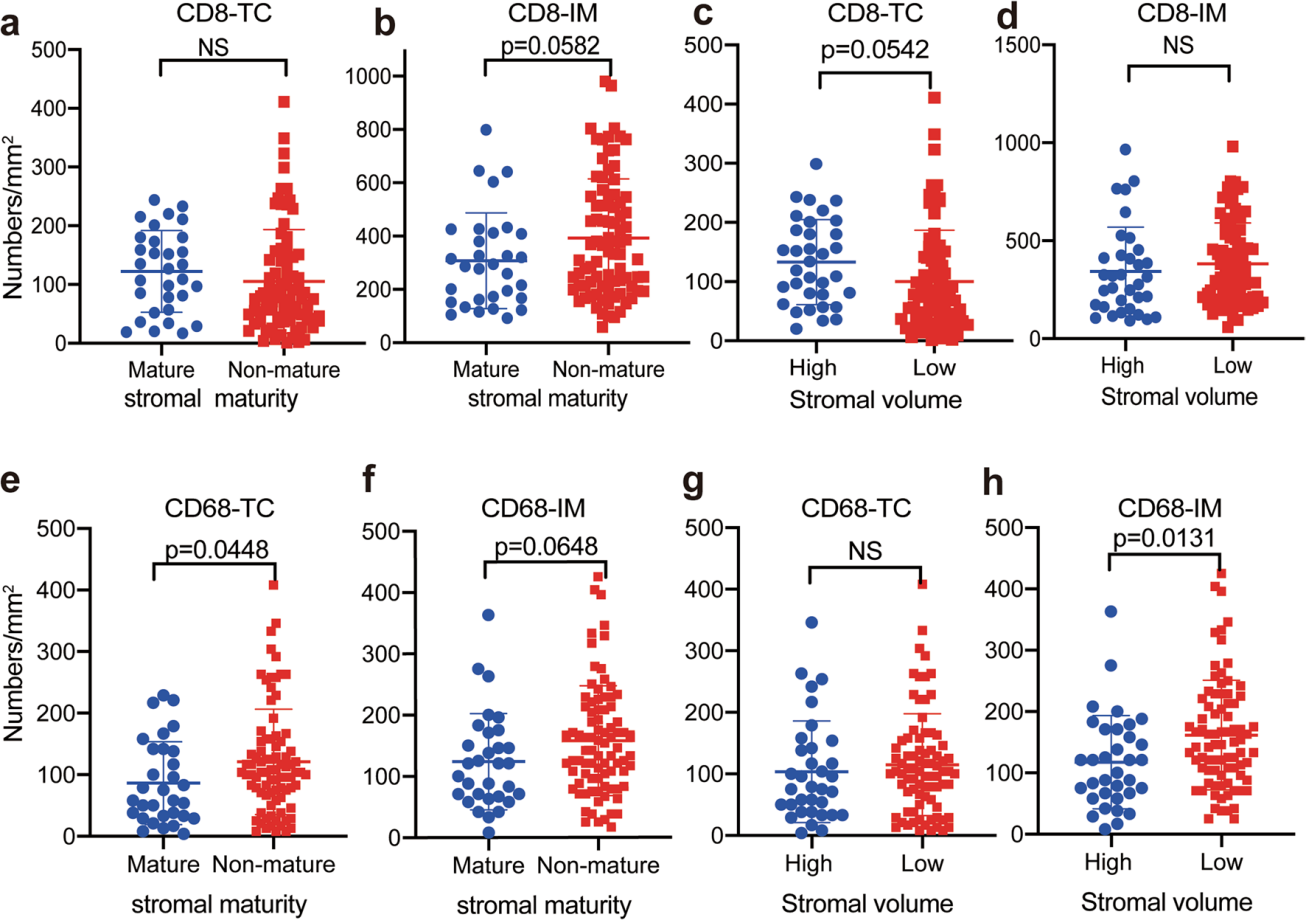
Relationships of tumor stromal volume and maturity with different locations of CD8^+^ and CD68^+^ cells in pancreatic ductal adenocarcinoma. Relationships of stromal maturity and CD8^+^ cells **a** at the TC and **b** at the IMs. Relationships of stromal volume and CD8^+^ cells **c** at the TC and **d** at the IMs. Relationships of stromal maturity and CD68^+^ cells **e** at the TC and **f** at the IMs. Relationships of stromal volume and CD68^+^ cells **g** at the TC and **h** at the IMs. *TC* tumor center, *IMs* invasive margins

Next, we analyzed the relationships between the stromal characteristics and the spatiotemporal distribution of infiltrating immune cells. When the cases were grouped into mature and non-mature stromal types, the results showed that tumors with a non-mature stroma had a trend toward more CD8_IM_^+^ (*p* = 0.0582, Fig. 4b), CD68_TC_^+^ (*p* = 0.0448, Fig. 4e), and CD68_IM_^+^ (*p* = 0.0646, Fig. 4f) cells. However, a higher stromal volume was correlated with more CD8_TC_^+^ (*p* = 0.0542, Fig. 4c) and less CD68_IM_^+^ (*p* = 0.0131, Fig. 4h) cells. No significant correlations were found between the stromal characteristics and the spatiotemporal distribution of CD4^+^ and CD206^+^ cells (Figure S4).

### Prognostic factors for OS in patients with PDAC

In terms of the relationships between the different immune cell locations and the clinicopathological characteristics of the patients, we found that a larger number of CD4_TC_^+^ cells was significantly associated with a lower serum carcinoembryonic antigen concentration (*p* = 0.0313), a larger number of CD8_TC_^+^ cells was associated with a lower histological grade (*p* = 0.0195), a lower number of CD68_IM_^+^ cells was associated with a lower number of TAIs (*p* = 0.0272) and a higher stromal volume (*p* = 0.0399), and a lower number of CD206_TC_^+^ cells was associated with a lower rate of tumor synchronous metastasis (*p* = 0.0109). However, no significant results were found among the other clinicopathological features (Tables S3–S6).

**Fig. 5:**
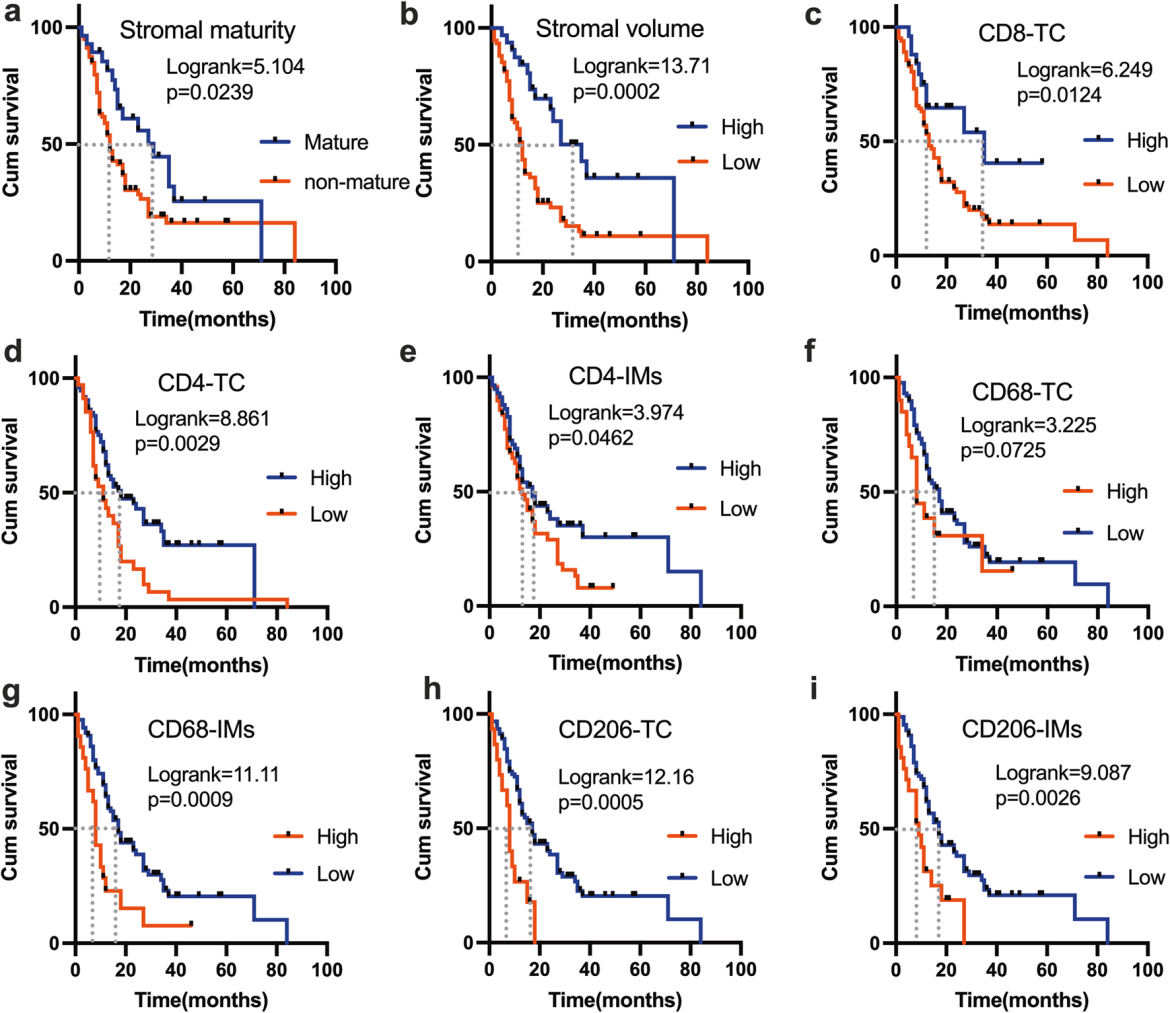
Prognostic factors for overall survival in patients with pancreatic ductal adenocarcinoma determined by the Kaplan–Meier analysis. **a** Stratification by the degree of stromal maturity. **b** Stratification by the tumor–stroma ratio. **c** Stratification by the quantity of CD8^+^ cells at the TC. **d** Stratification by the quantity of CD4^+^ cells at the TC. **e** Stratification by the quantity of CD4^+^ cells at the IMs. **f** Stratification by the quantity of CD68^+^ cells at the TC. **g** Stratification by the quantity of CD68^+^ cells at the IMs. **h** Stratification by the quantity of CD206^+^ cells at the TC. **i** Stratification by the quantity of CD206^+^ cells at the IMs. *TC* tumor center, *IMs* invasive margins

In terms of OS, the Kaplan–Meier survival analysis showed that patients with a mature stroma (log-rank = 5.104, *p* = 0.0239; Mature/Nonmature (MS): 29 vs 12; Fig. 5a); a higher stromal volume (log-rank = 13.71, *p* = 0.0002; high/low (MS):35 vs 12; Fig. 5b); and more CD8_TC_^+^ cells (log-rank = 6.249, *p* = 0.0124; high/low (MS):35vs13;Fig. 5c), CD4_TC_^+^cells (log-rank = 8.861,*p* = 0.0029;high/low (MS):18vs11; Fig. 5d), and CD4_IM_^+^ cells (log-rank = 3.974, *p* = 0.0462; high/low (MS):17vs13; Fig. 5e) had better PDAC outcomes. Conversely, patients with more CD68_IM_^+^ cells (log-rank = 11.11, *p* = 0.0009; high/low (MS):8 vs17; Fig. 5g), CD206_TC_^+^ cells (log-rank = 12.16, *p* = 0.0005;high/low (MS):8 vs17; Fig. 5h), and CD206_IM_^+^ cells (log-rank = 9.087, *p* = 0.0026; high/low (MS):9 vs17; Fig. 5i) had poor outcomes. However, more CD68_TC_^+^ cells exhibited a prognostic trend, with a log-rank *p-*value of 0.0725 (high/low (MS):8vs17; Fig. 5f). No significant association was found between the number of CD8_IM_^+^ cells and PDAC outcomes (high/low (MS):13vs18, Figure S5).

Subsequently, univariate and multivariate Cox regression analyses were performed on stromal features and tumor-infiltrating immune cells with the inclusion of multiple clinicopathological parameters of patients with PDAC. As shown in Table 2, TNM stage (*p* = 0.01), histological grade (*p* = 0.004), pN stage (*p* = 0.016), TSR (*p* < 0.001), tumor–stromal maturity (*p* = 0.025), CD4_TC_^+^ (*p* = 0.003), CD4_IM_^+^ (*p* = 0.046), CD8_TC_^+^ (*p* = 0.016), CD68_IM_^+^ (*p* = 0.001), CD206_TC_^+^ (*p* = 0.001), and CD206_IM_^+^ (*p* = 0.003) exhibited predictive significance. The multivariate analysis was then performed using only the features that were statistically significant in the univariate analysis. The results of the multivariate regression analysis showed that TNM stage (*p* = 0.0443, hazard ratio [HR] = 2.1, 95% confidence interval [CI] = 1.02–4.33), CD4_TC_^+^ (*p* = 0.0044, HR = 0.43, 95% CI = 0.24–0.77), CD8_TC_^+^ (*p* = 0.0129, HR = 0.36, 95% CI = 0.16–0.81), CD206_TC_^+^ (*p* = 0.0035, HR = 3.26, 95% CI = 1.47–7.22), and CD206_IM_^+^ (*p* = 0.0059, HR = 2.56, 95% CI = 1.31–4.98) were independent risk factors for patients with PDAC. The TSR (*p* = 0.0874, HR = 0.54, 95% CI = 0.26–1.1), CD4_IM_^+^ (*p* = 0.0657, HR = 0.61, 95% CI = 0.36–1.03), and CD68_IM_^+^ T cell number (*p* = 0.0657, HR = 1.87, 95% CI = 0.96–3.65) also demonstrated a trend toward predictive potential for PDAC. In general, our results demonstrated that tumor stromal features and the distinct spatial heterogeneity of immune cells may be useful in clinical practice to predict survival in patients with PDAC.

**Table 2:** Prognostic factors for the overall survival of patients with pancreatic ductal adenocarcinoma

Characteristics	Univariate analysis			Multivariate analysis		
	HR	95% CI	*p*-value	HR	95% CI	*p*-value
Age						
≤ 60 years	1					
> 60 years	1.32	0.84–2.09	0.229			
Sex						
Male	1					
Female	0.89	0.56–1.42	0.634			
TNM grade						
I + II	1			1		
III + IV	1.94	1.17–3.22	0.01	2.1	1.02–4.33	0.0443
Histological grade						
Good	1			1		
Moderate	1.58	0.77–3.25	0.216	0. 48	0.21–1.13	0.0931
Poor	2.48	1.33–4.61	0.004	1. 09	0.52–2.26	0.8225
Nerve invasion						
Absence	1					
Presence	1. 24	0. 75–2.05	0.403			
Extra-pancreatic invasion						
Absence	1					
Presence	1.18	0.62–2.24	0.622			
pT-stage						
T1	1					
T2	0.91	0.5–1.65	0.766			
T3	1.15	0.58–2.27	0.685			
pN-stage						
N1	1			1		
N2	1.42	0.87–2.31	0.162	1.42	0.83–2.44	0.2057
N3	2.4	1.17–4.93	0.016	0.7	0.25–1.96	0.4933
Serum CA125						
High	1					
Low	0.64	0.36–1.15	0.134			
Serum CA199						
High	1					
Low	0.82	0.45–1.51	0.528			
Serum CEA						
High	1					
Low	0.85	0.5–1.44	0.541			
Tumor–stroma ratio						
Stromal volume low	1			1		
Stromal volume high	0.36	0.21–0.63	< 0.001	0.54	0.26–1.1	0.0874
Stromal maturity						
Mature	1			1		
Non-mature	1.87	1.08–3.22	0.025	1.24	0.61–2.52	0.5442
CD4^+^ TC						
Low	1			1		
High	0.5	0.31–0.79	0.003	0.43	0.24–0.77	0.0044
CD4^+^ IMs						
Low	1			1		
High	0.63	0.4–0.99	0.046	0.61	0.36–1.03	0.0657
CD8^+^ TC						
Low	1			1		
High	0.43	0.21–0.85	0.016	0.36	0.16–0.81	0.0129
CD8^+^ IMs						
Low	1					
High	1.54	0.91–2.61	0.109			
CD68^+^ TC						
Low	1					
High	1.69	0.94–3.03	0.08			
CD68^+^ IMs						
Low	1			1		
High	2.46	1.43–4.22	0.001	1.87	0.96–3.65	0.0657
CD206^+^ TC						
Low	1			1		
High	2.92	1.56–5.44	0.001	3.26	1.47–7.22	0.0035
CD206^+^ IMs						
Low	1			1		
High	2.31	1.32–4.03	0.003	2.56	1.31–4.98	0.0059

*HR* hazard ratio, *CI* confidence interval, Non-mature includes intermediate and immature

### Development of a risk nomogram to predict OS in patients with PDAC

**Fig. 6:**
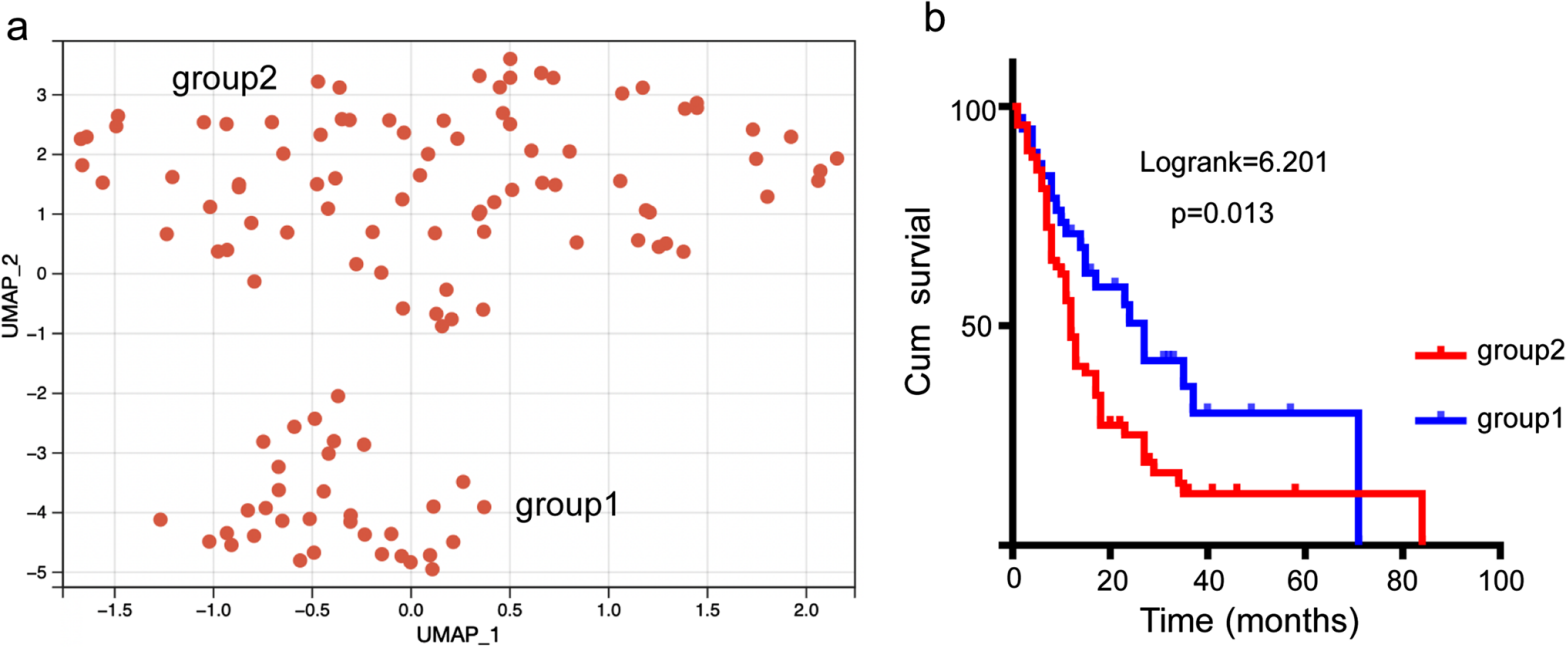
UMAP dimension reduction analysis in patients with pancreatic ductal adenocarcinoma. **a** Illustrations of the groups defined by the UMAP analysis. **b** Kaplan–Meier survival analysis for patients divided by UMAP dimension reduction

**Fig. 7:**
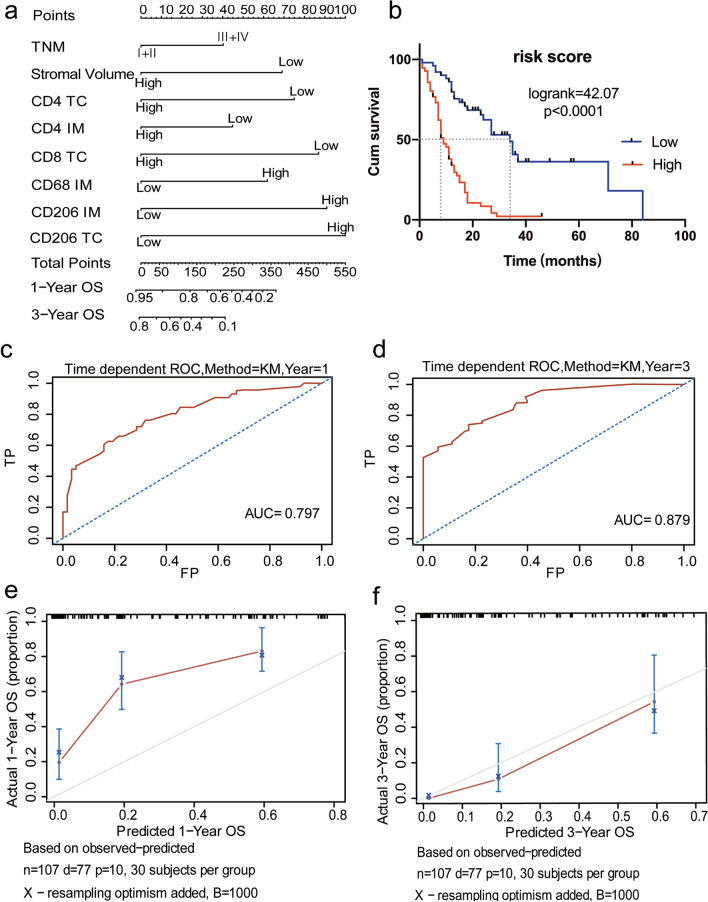
Prognostic nomogram for patients with pancreatic ductal adenocarcinoma (PDAC). **a** Nomogram to predict the 1-year and 3-year overall survival of patients with PDAC. Mark patient values at each axis, draw a straight line perpendicular to the point axis, and sum the points for all variables. Next, mark the sum on the total point axis and draw a straight line perpendicular to the probability axis. **b** Kaplan–Meier survival analysis for patients with a model-predicted risk score. The median risk score (280) was used as the cut-off value. **c** Receiver operating characteristic (ROC) curve for the prediction at 1 year. **d** ROC curve for the prediction at 3 years. **e** The calibration curve of the nomogram for predicting OS at 1 year. The x-axis shows the predicted probability of death from PDAC. The y-axis shows the observed probability of death from PDAC. The dashed diagonal line represents the ideal nomogram, and the blue and red lines represent the 1-year nomograms. **f** The calibration curve of the nomogram for predicting OS at 3 years. The x-axis shows the predicted probability of death from PDAC. The y-axis shows the observed probability of death from PDAC. The dashed diagonal line represents the ideal nomogram, and the blue and red lines represent the 3-year nomograms

For easy use in clinical management, we established a risk nomogram to predict the survival probability of patients with PDAC when including the parameters with a *p* value of less than 0.1 from the multivariate Cox regression analysis. First, UMAP was performed to verify the effectiveness of the parameters. The results showed two distinct patient clusters (Fig. 6a). The Kaplan–Meier survival analysis showed that the outcomes of the two groups were significantly different (log-rank = 6.201, *p* = 0.013;group1/group2 (MS):27vs12; Fig. 6b). The included parameters are shown in Fig. 7a. The parameters were binary variables, and the score of each factor corresponds to the point bar at the top of the table. The total score for each patient was added one by one into the nomogram, which was associated with the OS probability at 1 and 3 years. For instance, a patient with a TNM stage of II, a lower stromal volume, a higher CD4_TC_^+^ T cell number, a higher CD4_IM_^+^ T cell number, a lower CD8_TC_^+^ T cell number, a lower CD68_IM_^+^ macrophage number, a lower CD206_TC_^+^ macrophage number, and a lower CD206_IM_^+^ macrophage number would generate a total of 150 points, which indicated a 1-year OS rate of 70% and a 3-year OS rate of 50% for this patient.

To explore the predictive efficiency of this risk nomogram, we performed the Kaplan–Meier analysis by stratifying all patients according to the median risk scores derived from the risk nomogram. Using the median as the cut-off value, we found that patients with a low risk score exhibited substantially better outcomes than patients with a high risk score (*p* < 0.0001, HR = 0.1863, 95% CI = 0.1617–0.4161, log-rank = 42.07; high/low (MS): 9vs34) (Fig. 7b). Moreover, our prediction model was further validated by computing the discrimination index (the Harrell concordance index [c-index]) and calibration curves of the 1-year and 3-year OS. After bootstrap resampling 1000 times for internal validation, the results showed that the c-index was 0.772 (95% CI = 0.713–0.831) in the risk nomogram. The area under the ROC curve of the calibration curves for 1-year and 3-year OS were 0.797 and 0.879, respectively (Fig. 7c, d), with good agreement between the predicted survival probabilities and the observed outcomes (Fig. 7e, f).

## Discussion

The limitations of traditional prognostic tools in clinical practice suggest that it require some improvements in terms of treatment strategies in PDAC [31]. In the presented study, we found that eight parameters, including TNM stage; TSR; and the quantities of CD4TC + , CD4IM + , CD8TC + , CD68IM + , CD206TC + , and CD206IM + cells had a predictive effect on the prognosis of patients with PDAC. A risk nomogram that integrates the characteristics of the tumor and the TME would stratify patients into low and high risk groups, which will facilitate clinical management. However, the clear functions and interactions between immune cells and stromal features remain unknown.

Except for tumor burden, components of TME, particularly for tumor stroma and tumor infiltrating immune cells exert important roles in the progression of PDAC. Similar with the previously studies [13], TME of PDAC exhibited great heterogeneity both in the histomorphologic characteristics and their substantial compartments across cases in our presented study.It is well known that CAFs are important components of the TME that exhibit morphological and functional changes when was activated [13]. When we assessed stromal maturity according to the percentage of activated CAFs, we observed that non-mature (intermediate and immature) stromal type was associated with lower stromal volume and a higher number of TAIs, particularly in terms of increasing CD68 positive macrophages infiltrating in the whole tumor. When regarding the spatiotemporal distribution of immune cells, this non-mature stroma had a trend with more CD68 + cells both in IMs and TC and CD8 + cells in IMs. Interestingly, higher stromal volume was correlated with more CD8 + cells in TC and lower CD68 + cells in IMs. In addition, we also found the presence of non-mature stroma and lower stromal volume had negative effect on survival in PDAC patients. Beyond tumor cells, our presented data indicated that the stromal maturity and TSR showed the relationships with the tumor infiltrating immune cells, suggesting their roles in regulating the TME and resulting in the malignant progression of PDAC. It is reported that CAFs could act on CD8 + T cells and F4/80 macrophages directly in the process of ECM remodelling [32–34]. Immature stroma was dominated by activated CAFs and is deficient in mature collagen, which may offer an anoxic and acidic microenvironment to inhibit CD8 positive T cells migration and otherwise promote the recruitment macrophages [32, 35]. With the technology of single cell sequencing, Grünwald BT et al. found the functional molecular properties were enriched in inflammation-related pathways in the subgroup of reactive “subTMEs” [36]. Stroma derived CAFs could directly kill CD8 + T cells through PD-L2 and FASL in an antigen-dependent manner [37]. Thus, targeting the stroma could induce changes in the density and distribution of immune cells in PDAC, and further study is warranted to investigate this possibility.

The distribution of infiltrating immune cells exhibited great spatial heterogeneity. In the present study, instead of evaluating the separate regions of the TC and IMs using the whole-tissue PDAC sections, we found that subsets of T lymphocytes, pan-macrophages, and M2 macrophages in IM regions were significantly more abundant than those in TC regions, suggesting that the majority of the PDAC is an altered or cold immune tumor [38]. Tumor-infiltrating immune cells in the TC and IMs showed close associations with each other. CD4^+^ T cells were significantly positively correlated with CD8^+^, CD68^+^, and CD206^+^ cells. It has been indicated that CD4^+^ T cells play a central role in orchestrating the host immune response against cancer in animal models [21]. However, CD4^+^ T cells are not a single cell population; instead, they include T helper cells and regulatory T cells, which demonstrate anti-tumor immune responses and reinforce tumor immune tolerance at the tumor site [39]. Thus, it is reasonable to speculate that CD4^+^ T cells may play a role in the early stage of PDAC development, subsequently recruiting effector CD8^+^ T cells and macrophages to the tumor site to maintain the balance of the TME.

TAMs and T lymphocytes are important cell populations that generally exhibit a double-edged sword of immune effects in the context of cancer [40]. TAMs are associated with a poor prognosis in many types of cancer, which is due, in part, to the production of various factors that promote angiogenesis and tissue invasion [41]. T cells are generally considered as the key fighters in the antitumor immunoreaction, and they help to guide treatment selection in multiple cancers. However, partly due to the diversity in the methods used in different PDAC studies, the prognostic value of T cell subsets still remains controversial. In the present study, we found that the density of TAMs and T lymphocytes, as well as their spatial distribution, had prognostic value for PDAC, as has previously been demonstrated in other cancers [42, 43]. Infiltrating immune cells in the TC exhibited more powerful prognostic significance than those in the IMs, which also supports the opinion that the distance between tumor cells and infiltrating immune cells is a key factor in determining their ability to attack or suppress the tumor via their cell–cell contact or paracrine effects [44]. For T cell subsets, similar to CD8^+^ T cells at the TC, CD4^+^ T cells exhibited anti-tumor effects and prolonged the OS of patients with PDAC. It has been reported previously that activated CD4^+^ T cells become cytotoxic and kill major histocompatibility complex-II^+^ PDAC cells as efficiently as CD8^+^ T cells [45]. Our data suggest that CD4^+^ T cells contribute to better outcomes in patients with PDAC, which may be mediated through their direct cytotoxicity against tumor cells and action as helper cells to potentiate dendritic cells, resulting in enhanced CD8^+^ T cell responses [21]. A previous study showed that CD8^+^ T cells become trapped within the peri-tumorous, resulting in disable to attack the cancer cells [34]. Thus, targeted therapy to induce the migration of cytotoxic T lymphocytes to the TC is important. A recently research of PDAC exhibited that spatial correlations using spatial G(r)function names (Gcross) values were significantly higher in long-term survivors for directions between CD4^+^ T cells and myelomonocytes, while only Gcross (B cell-CD8^+^Tcells) was significantly higher in short-term survivors. It indicated that CD4^+^ T cells co-exhibited with myelomonocytes could benefit to patients with long-term survival, while short-term survivors tended to reflect increased density of CD8^+^T cells in areas containing B cells [46]. Thus, for future clinical application, in addition to T cells, which were suggested in the model of colorectal cancer immunoscore, other types of immune cell would be proposed as regarding to the different malignancies [47]. Furthermore, more subtle metrics would evaluate the great spatial heterogeneity of TME at multiscale levels in PDAC, which could help to discern subtle biological differences between patients with poor and improved survival in future [48].

This study has some limitations that should be noted. First, although integrated TNM staging and features of the TME can determine OS in patients with PDAC, further validation should be explored in a larger independent cohort or in a multicentric study. More and valuable results could be taken into account when it confers to poor prognostic outcome of PDAC. Second, limitations of the materials used precluded a precise delineation of the tumor and the IMs; thus, the results should be further validated before they can be applied clinically. Finally, the immune features studied were incomplete and did not include factors such as CD20, CD3, FoxP3, which may have limited the results.

## Conclusion

In conclusion, we found that stromal features, including stromal maturity and TSR, were closely associated with TAI infiltration in the stroma, particularly in terms of the density and location of CD68^+^ macrophages and cytotoxic CD8^+^ T cells. The subsets of T lymphocytes, pan-macrophages, and M2 macrophages in IMs were significantly more abundant than those in the TC, and the infiltrating cells in the TC had more powerful prognostic significance than those in the IMs. Combining TNM staging and TME parameters could help to provide a more individualized prognostic prediction in patients with PDAC. However, further validation in a larger independent cohort or in a multicentric study could be taken into account.

## Supplementary Information

Supplementary file 1: Table S1. The clinicalpathology features of 116 PDAC patients. TableS2.The median survivals for each group. Tables S3–S6. Associations between the locations of immune cells and clinicopathological characteristics in pancreatic ductal adenocarcinoma. Figure S1. The relationships of stromal maturity and the TSR with CD4+, CD8+, and CD206+ cells in whole slides. Figure S2. Distribution of infiltrating immune cells and the most optimal cut-off value for CD4 and CD8 positive lymphocytes . Figure S3. The distribution of infiltrating immune cells and the most optimal cut-off value for CD68 and CD206 positive macrophages. Figure S4. Relationships of tumor stromal volume and maturity with different locations of CD4+ and CD206+ cells in pancreatic ductal adenocarcinoma. Figure S5. Kaplan–Meier survival analysis of patients with different CD8+ immune cell quantities at the IMs.

## Data Availability

All of the data supporting the results are shown in the paper.
