# Supplementary material for: The tumor–stroma ratio and the immune microenvironment improve the prognostic prediction of pancreatic ductal adenocarcinoma
Source: Discov Oncol. 2023 Jul 5;14:124. doi: 10.1007/s12672-023-00744-w (PMC10322806; doi:10.1007/s12672-023-00744-w)
Supplement: Supplementary file 1 — Table S1. The clinicalpathology features of 116 PDAC patients. TableS2.The median survivals for each group. Tables S3–S6. Associations between the locations of immune cells and clinicopathological characteristics in pancreatic ductal adenocarcinoma. Figure S1. The relationships of stromal maturity and the TSR with CD4+, CD8+, and CD206+ cells in whole slides. Figure S2. Distribution of infiltrating immune cells and the most optimal cut-off value for CD4 and CD8 positive lymphocytes . Figure S3. The distribution of infiltrating immune cells and the most optimal cut-off value for CD68 and CD206 positive macrophages. Figure S4. Relationships of tumor stromal volume and maturity with different locations of CD4+ and CD206+ cells in pancreatic ductal adenocarcinoma. Figure S5. Kaplan–Meier survival analysis of patients with different CD8+ immune cell quantities at the IMs. [file 12672_2023_744_MOESM1_ESM.docx]

| Table S1.The clinicalpathology features of 116 PDAC patients | | | |  |
| --- | --- | --- | --- | --- |
| characteristics | dead(N=77) | live(N=30) | lost(N=9) | Overall(N=116) |
| Size |  |  |  |  |
| Mean (SD) | 3.66 (1.89) | 3.70 (1.49) | 3.48 (2.10) | 3.66 (1.80) |
| Median [Min, Max] | 3.30 [0.700, 11.0] | 3.20 [1.70, 7.00] | 2.90 [0.900, 7.20] | 3.30 [0.700, 11.0] |
| Age |  |  |  |  |
| Mean (SD) | 1.55 (0.501) | 1.47 (0.507) | 1.56 (0.527) | 1.53 (0.501) |
| Median [Min, Max] | 2.00 [1.00, 2.00] | 1.00 [1.00, 2.00] | 2.00 [1.00, 2.00] | 2.00 [1.00, 2.00] |
| Gender |  |  |  |  |
| Female | 29 (37.7%) | 14 (46.7%) | 3 (33.3%) | 46 (39.7%) |
| Male | 48 (62.3%) | 16 (53.3%) | 6 (66.7%) | 70 (60.3%) |
| Pt |  |  |  |  |
| T1 | 15 (19.5%) | 2 (6.7%) | 2 (22.2%) | 19 (16.4%) |
| T2 | 42 (54.5%) | 18 (60.0%) | 5 (55.6%) | 65 (56.0%) |
| T3 | 20 (26.0%) | 10 (33.3%) | 2 (22.2%) | 32 (27.6%) |
| N |  |  |  |  |
| N0 | 33 (42.9%) | 18 (60.0%) | 4 (44.4%) | 55 (47.4%) |
| N1 | 34 (44.2%) | 11 (36.7%) | 3 (33.3%) | 48 (41.4%) |
| N2 | 10 (13.0%) | 1 (3.3%) | 2 (22.2%) | 13 (11.2%) |
| M |  |  |  |  |
| M0 | 69 (89.6%) | 30 (100%) | 9 (100%) | 108 (93.1%) |
| M1 | 8 (10.4%) | 0 (0%) | 0 (0%) | 8 (6.9%) |
| TNM |  |  |  |  |
| I+II | 55 (71.4%) | 24 (80.0%) | 7 (77.8%) | 86 (74.1%) |
| III+IV | 22 (28.6%) | 6 (20.0%) | 2 (22.2%) | 30 (25.9%) |
| Histological grade |  |  |  |  |
| Well | 13 (16.9%) | 15 (50.0%) | 2 (22.2%) | 30 (25.9%) |
| Moderate | 18 (23.4%) | 3 (10.0%) | 3 (33.3%) | 24 (20.7%) |
| Poor | 46 (59.7%) | 12 (40.0%) | 4 (44.4%) | 62 (53.4%) |
| serum CA125 (U/L) |  |  |  |  |
| High | 16 (20.8%) | 5 (16.7%) | 2 (22.2%) | 23 (19.8%) |
| Low | 45 (58.4%) | 20 (66.7%) | 3 (33.3%) | 68 (58.6%) |
| Missing | 16 (20.8%) | 5 (16.7%) | 4 (44.4%) | 25 (21.6%) |
| serum CA199 (U/L) |  |  |  |  |
| High | 56 (72.7%) | 20 (66.7%) | 5 (55.6%) | 81 (69.8%) |
| Low | 13 (16.9%) | 9 (30.0%) | 2 (22.2%) | 24 (20.7%) |
| Missing | 8 (10.4%) | 1 (3.3%) | 2 (22.2%) | 11 (9.5%) |
| serum CEA (ng/ml) |  |  |  |  |
| High | 22 (28.6%) | 9 (30.0%) | 2 (22.2%) | 33 (28.4%) |
| Low | 42 (54.5%) | 18 (60.0%) | 5 (55.6%) | 65 (56.0%) |
| Missing | 13 (16.9%) | 3 (10.0%) | 2 (22.2%) | 18 (15.5%) |
|  | | | | |
| Continued Table S1.The clinicalpathology features of 116 PDAC patients | | | | |
| characteristics | dead(N=77) | live(N=30) | lost(N=9) | Overall(N=116) |
| Tumor stromal volume | | | | |
| Low | 61 (79.2%) | 14 (46.7%) | 7 (77.8%) | 82 (70.7%) |
| High | 16 (20.8%) | 16 (53.3%) | 2 (22.2%) | 34 (29.3%) |
| Maturity |  |  |  |  |
| Mature | 17 (22.1%) | 11 (36.7%) | 3 (33.3%) | 31 (26.7%) |
| Moderate | 21 (27.3%) | 8 (26.7%) | 3 (33.3%) | 32 (27.6%) |
| Immature | 39 (50.6%) | 11 (36.7%) | 3 (33.3%) | 53 (45.7%) |
| CD4 TC |  |  |  |  |
| Mean (SD) | 55.7 (95.9) | 66.9 (62.5) | 46.9 (45.3) | 57.9 (85.1) |
| Median [Min, Max] | 29.0 [4.00, 572] | 49.0 [6.00, 268] | 18.0 [7.00, 131] | 32.0 [4.00, 572] |
| CD8 TC |  |  |  |  |
| Mean (SD) | 98.7 (74.3) | 151 (101) | 72.0 (48.5) | 110 (83.8) |
| Median [Min, Max] | 80.0 [2.00, 350] | 161 [5.00, 411] | 75.0 [14.0, 143] | 86.5 [2.00, 411] |
| CD68 TC |  |  |  |  |
| Mean (SD) | 105 (84.6) | 127 (80.4) | 121 (69.0) | 112 (82.4) |
| Median [Min, Max] | 84.0 [9.00, 409] | 107 [5.00, 346] | 117 [34.0, 263] | 100 [5.00, 409] |
| CD206 TC |  |  |  |  |
| Mean (SD) | 57.9 (59.5) | 56.1 (37.4) | 57.8 (71.8) | 57.4 (55.2) |
| Median [Min, Max] | 34.0 [0, 275] | 55.0 [5.00, 150] | 38.0 [5.00, 238] | 38.0 [0, 275] |
| CD4 IM |  |  |  |  |
| Mean (SD) | 326 (251) | 416 (225) | 305 (291) | 348 (249) |
| Median [Min, Max] | 275 [15.0, 1480] | 370 [56.0, 947] | 196 [4.00, 847] | 284 [4.00, 1480] |
| CD8 IM |  |  |  |  |
| Mean (SD) | 363 (209) | 400 (232) | 337 (214) | 371 (214) |
| Median [Min, Max] | 305 [59.0, 980] | 353 [99.0, 966] | 313 [123, 799] | 318 [59.0, 980] |
| CD68 IM |  |  |  |  |
| Mean (SD) | 155 (95.9) | 137 (70.2) | 135 (65.6) | 149 (87.8) |
| Median [Min, Max] | 125 [17.0, 425] | 140 [9.00, 275] | 163 [25.0, 225] | 134 [9.00, 425] |
| CD206 IM |  |  |  |  |
| Mean (SD) | 94.7 (71.3) | 85.4 (38.2) | 60.2 (55.7) | 89.6 (63.5) |
| Median [Min, Max] | 75.0 [9.00, 400] | 80.0 [9.00, 163] | 46.0 [9.00, 188] | 75.0 [9.00, 400] |

| TableS2.The median survivals for each group | |
| --- | --- |
| Variables | Median survival（month） |
| CD4-TC |  |
| low | 11 |
| high | 18 |
| CD4-IM |  |
| low | 13 |
| high | 17 |
| CD8-TC |  |
| low | 13 |
| high | 35 |
| CD8-IM |  |
| low | 18 |
| high | 13 |
| CD68-TC |  |
| low | 17 |
| high | 8 |
| CD68-IM |  |
| low | 17 |
| high | 8 |
| CD206-TC |  |
| low | 17 |
| high | 8 |
| CD206-IM |  |
| low | 17 |
| high | 9 |
| TSR |  |
| low | 35 |
| high | 12 |
| maturity |  |
| mature | 29 |
| non-mature | 12 |

Table S3 Associations between the locations of CD4+ immune cells and clinicopathological characteristics in pancreatic ductal adenocarcinoma

| characteristics | CD4 TC | | *p*-value | CD4 IMs | | *p*-value |
| --- | --- | --- | --- | --- | --- | --- |
|  | Low (n=37) | High(n=79) |  | Low (n=56) | High (n=60) |  |
| Age |  |  |  |  |  |  |
| <60 years | 17 (45.95) | 38 (48.10) | 0.9863 | 26 (46.43) | 29 (48.33) | 0.9846 |
| ≥60 years | 20 (54.05) | 41 (51.90) |  | 30 (53.57) | 31 (51.67) |  |
| Sex |  |  |  |  |  |  |
| Male | 23 (62.16) | 47 (59.49) | 0.944 | 31 (55.36) | 39 (65.00) | 0.3838 |
| Female | 14 (37.84) | 32 (40.51) |  | 25 (44.64) | 21 (35.00) |  |
| pT stage |  |  |  |  |  |  |
| T1 | 7 (18.92) | 12 (15.19) | 0.1444 | 9 (16.07) | 10 (16.67) | 0.8086 |
| T2 | 16 (43.24) | 49 (62.03) |  | 30 (53.57) | 35 (58.33) |  |
| T3 | 14 (37.84) | 18 (22.78) |  | 17 (30.36) | 15 (25.00) |  |
| pN stage |  |  |  |  |  |  |
| N0 | 15 (40.54) | 40 (50.63) | 0.5848 | 23 (41.07) | 32 (53.33) | 0.1958 |
| N1 | 17 (45.95) | 31 (39.24) |  | 24 (42.86) | 24 (40.00) |  |
| N2 | 5 (13.51) | 8 (10.13) |  | 9 (16.07) | 4 (6.67) |  |
| M stage |  |  |  |  |  |  |
| M0 | 33 (89.19) | 75 (94.94) | 0.456 | 51 (91.07) | 57 (95.00) | 0.6399 |
| M1 | 4 (10.81) | 4 (5.06) |  | 5 (8.93) | 3 (5.00) |  |
| TNM grade |  |  |  |  |  |  |
| I+II | 25 (67.57) | 61 (77.22) | 0.3797 | 39 (69.64) | 47 (78.33) | 0.392 |
| III+IV | 12 (32.43) | 18 (22.78) |  | 17 (30.36) | 13 (21.67) |  |
| Histological grade |  |  |  |  |  |  |
| Good | 6 (16.22) | 24 (30.38) | 0.2633 | 10 (17.86) | 20 (33.33) | 0.1108 |
| Moderate | 9 (24.32) | 15 (18.99) |  | 11 (19.64) | 13 (21.67) |  |
| Poor | 22 (59.46) | 40 (50.63) |  | 35 (62.50) | 27 (45.00) |  |
| Tumor associated immune cells |  |  |  |  |  |  |
| Low | 20 (54.05) | 44 (55.70) | 1 | 33 (58.93) | 31 (51.67) | 0.5491 |
| High | 17 (45.95) | 35 (44.30) |  | 23 (41.07) | 29 (48.33) |  |
| SerumCA125 |  |  |  |  |  |  |
| High | 10 (35.71) | 13 (20.63) | 0.2054 | 15 (34.88) | 8 (16.67) | 0.0793 |
| Low | 18 (64.29) | 50 (79.37) |  | 28 (65.12) | 40 (83.33) |  |
| SerumCEA |  |  |  |  |  |  |
| High | 16 (50.00) | 17 (25.76) | 0.0313 | 21 (43.75) | 12 (24.00) | 0.0637 |
| Low | 16 (50.00) | 49 (74.24) |  | 27 (56.25) | 38 (76.00) |  |
| SerumCA199 |  |  |  |  |  |  |
| High | 24 (72.73) | 57 (79.17) | 0.6318 | 36 (73.47) | 45 (80.36) | 0.5448 |
| Low | 9 (27.27) | 15 (20.83) |  | 13 (26.53) | 11 (19.64) |  |

Continued Table S3 Associations between the locations of CD4+ immune cells and clinicopathological characteristics in pancreatic ductal adenocarcinoma

| characteristics | CD4 TC | | *p*-value | CD4 IMs | | *p*-value |
| --- | --- | --- | --- | --- | --- | --- |
|  | Low (n=37) | High(n=79) |  | Low (n=56) | High (n=60) |  |
| Nerve Invasion |  |  |  |  |  |  |
| Absence | 9 (24.32) | 24 (30.38) | 0.6506 | 17 (30.36) | 16 (26.67) | 0.8147 |
| Presence | 28 (75.68) | 55 (69.62) |  | 39 (69.64) | 44 (73.33) |  |
| Extra-pancreatic Invasion |  |  |  |  |  |  |
| Absence | 5 (13.51) | 12 (15.19) | 1 | 7 (12.50) | 10 (16.67) | 0.7103 |
| Presence | 32 (86.49) | 67 (84.81) |  | 49 (87.50) | 50 (83.33) |  |
| Tumor-stroma ratio |  |  |  |  |  |  |
| Stromal low | 28 (75.68) | 54 (68.35) | 0.5562 | 42 (75.00) | 40 (66.67) | 0.4347 |
| Sromal high | 9 (24.32) | 25 (31.65) |  | 14 (25.00) | 20 (33.33) |  |
| Stroma maturity |  |  |  |  |  |  |
| Mature | 10 (27.03) | 21 (26.58) | 0.9215 | 16 (28.57) | 15 (25.00) | 0.8131 |
| Moderate | 11 (29.73) | 21 (26.58) |  | 14 (25.00) | 18 (30.00) |  |
| Immature | 16 (43.24) | 37 (46.84) |  | 26 (46.43) | 27 (45.00) |  |

Table S4 Associations between the locations of CD8+ immune cells and clinicopathological characteristics in pancreatic ductal adenocarcinoma

| characteristics | CD8 TC | | *p*-value | CD8 IMs | | *p*-value |
| --- | --- | --- | --- | --- | --- | --- |
|  | Low (n=93) | High(n=23) |  | Low(n=31) | High(n=85) |  |
| Age |  |  |  |  |  |  |
| <60 years | 43 (46.24) | 12 (52.17) | 0.7815 | 12 (38.71) | 43 (50.59) | 0.3556 |
| ≥60 years | 50 (53.76) | 11 (47.83) |  | 19 (61.29) | 42 (49.41) |  |
| Sex |  |  |  |  |  |  |
| Male | 53 (56.99) | 17 (73.91) | 0.2122 | 18 (58.06) | 52 (61.18) | 0.9293 |
| Female | 40 (43.01) | 6 (26.09) |  | 13 (41.94) | 33 (38.82) |  |
| pT stage |  |  |  |  |  |  |
| T1 | 17 (18.28) | 2 (8.70) | 0.4749 | 7 (22.58) | 12 (14.12) | 0.4995 |
| T2 | 50 (53.76) | 15 (65.22) |  | 17 (54.84) | 48 (56.47) |  |
| T3 | 26 (27.96) | 6 (26.09) |  | 7 (22.58) | 25 (29.41) |  |
| pN stage |  |  |  |  |  |  |
| N0 | 44 (47.31) | 11 (47.83) | 0.5268 | 14 (45.16) | 41 (48.24) | 0.8694 |
| N1 | 40 (43.01) | 8 (34.78) |  | 14 (45.16) | 34 (40.00) |  |
| N2 | 9 (9.68) | 4 (17.39) |  | 3 (9.68) | 10 (11.76) |  |
| M stage |  |  |  |  |  |  |
| M0 | 84 (92.31) | 22 (96.45) | 0.8417 | 31 (100.00) | 77 (90.59) | 0.175 |
| M1 | 7 (7.69) | 1 (3.55) |  | 0 (0.00) | 8 (9.41) |  |
| TNM grade |  |  |  |  |  |  |
| I+II | 70 (75.27) | 16 (69.57) | 0.7692 | 25 (80.65) | 61 (71.76) | 0.4672 |
| III+IV | 23 (24.73) | 7 (30.43) |  | 6 (19.35) | 24 (28.24) |  |
| Histological grade |  |  |  |  |  |  |
| High | 19 (20.43) | 11 (47.83) | 0.0195 | 7 (22.58) | 23 (27.06) | 0.8264 |
| Moderate | 22 (23.66) | 2 (8.70) |  | 6 (19.35) | 18 (21.18) |  |
| Poor | 52 (55.91) | 10 (43.48) |  | 18 (58.06) | 44 (51.76) |  |
| Tumor associated immune cells |  |  |  |  |  |  |
| Low | 53 (56.99) | 11 (47.83) | 0.5775 | 17 (54.84) | 47 (55.29) | 1 |
| High | 40 (43.01) | 12 (52.17) |  | 14 (45.16) | 38 (44.71) |  |
| SerumCA125 |  |  |  |  |  |  |
| High | 19 (27.14) | 4 (19.05) | 0.6438 | 8 (29.63) | 15 (23.44) | 0.7212 |
| Low | 51 (72.86) | 17 (80.95) |  | 19 (70.37) | 49 (76.56) |  |
| SerumCEA |  |  |  |  |  |  |
| High | 23 (29.87) | 10 (47.62) | 0.2058 | 13 (46.43) | 20 (28.57) | 0.1462 |
| Low | 54 (70.13) | 11 (52.38) |  | 15 (53.57) | 50 (71.43) |  |
| SerumCA199 |  |  |  |  |  |  |
| High | 62 (74.70) | 19 (86.36) | 0.3827 | 22 (75.86) | 59 (77.63) | 1 |
| Low | 21 (25.30) | 3 (13.64) |  | 7 (24.14) | 17 (22.37) |  |

Continued Table S4 Associations between the locations of CD8+ immune cells and clinicopathological characteristics in pancreatic ductal adenocarcinoma

| characteristics | CD8 TC | | *p*-value | CD8 IMs | | *p*-value |
| --- | --- | --- | --- | --- | --- | --- |
|  | Low (n=93) | High(n=23) |  | Low(n=31) | High(n=85) |  |
| Nerve Invasion |  |  |  |  |  |  |
| Absence | 29 (31.18) | 6 (26.09) | 0.4198 | 8 (25.81) | 25 (29.41) | 0.8821 |
| Presence | 64 (68.82) | 17 (73.91) |  | 23 (74.19) | 60 (70.59) |  |
| Extra-pancreatic Invasion |  |  |  |  |  |  |
| Absence | 12 (12.90) | 5 (21.74) | 0.4571 | 3 (9.68) | 14 (16.47) | 0.536 |
| Presence | 81 (87.10) | 18 (78.26) |  | 28 (90.32) | 71 (83.53) |  |
| Tumor-stroma ratio |  |  |  |  |  |  |
| Stromal low | 69 (74.19) | 13 (56.52) | 0.1581 | 20 (64.52) | 62 (72.94) | 0.5146 |
| Sromal high | 24 (25.81) | 10 (43.48) |  | 11 (35.48) | 23 (27.06) |  |
| Stroma maturity |  |  |  |  |  |  |
| Mature | 24 (25.81) | 7 (30.43) | 0.2237 | 12 (38.71) | 19 (22.35) | 0.1933 |
| Moderate | 23 (24.73) | 9 (39.13) |  | 8 (25.81) | 24 (28.24) |  |
| Immature | 46 (49.46) | 7 (30.43) |  | 11 (35.48) | 42 (49.41) |  |

Table S5 Associations between the locations of CD68+ immune cells and clinicopathological characteristics in pancreatic ductal adenocarcinoma

| characteristics | CD68 TC | | *p*-value | CD68 IMs | | *p*-value |
| --- | --- | --- | --- | --- | --- | --- |
|  | Low(n=94) | High(n=22) |  | Low(n=94) | High(n=22) |  |
| Age |  |  |  |  |  |  |
| <60 years | 42 (44.68) | 13 (59.09) | 0.3264 | 46 (48.94) | 9 (40.91) | 0.6588 |
| ≥60 years | 52 (55.32) | 9 (40.91) |  | 48 (51.06) | 13 (59.09) |  |
| Sex |  |  |  |  |  |  |
| Male | 59 (62.77) | 11 (50.00) | 0.3899 | 59 (62.77) | 11 (50.00) | 0.3899 |
| Female | 35 (37.23) | 11 (50.00) |  | 35 (37.23) | 11 (50.00) |  |
| pT stage |  |  |  |  |  |  |
| T1 | 17 (18.09) | 2 (9.09) | 0.4384 | 16 (17.02) | 3 (13.64) | 0.7272 |
| T2 | 53 (56.38) | 12 (54.55) |  | 51 (54.26) | 14 (63.64) |  |
| T3 | 24 (25.53) | 8 (36.36) |  | 27 (28.72) | 5 (22.73) |  |
| pN stage |  |  |  |  |  |  |
| N0 | 45 (47.87) | 10 (45.45) | 0.8872 | 46 (48.94) | 9 (40.91) | 0.4911 |
| N1 | 38 (40.43) | 10 (45.45) |  | 39 (41.49) | 9 (40.91) |  |
| N2 | 11 (11.70) | 2 (9.09) |  | 9 (9.57) | 4 (18.18) |  |
| M stage |  |  |  |  |  |  |
| M0 | 88 (93.62) | 20 (90.91) | 1 | 88 (93.62) | 20 (90.91) | 1 |
| M1 | 6 (6.38) | 2 (9.09) |  | 6 (6.38) | 2 (9.09) |  |
| TNM grade |  |  |  |  |  |  |
| I+II | 70 (74.47) | 16 (72.73) | 1 | 70 (74.47) | 16 (72.73) | 1 |
| III+IV | 24 (25.53) | 6 (27.27) |  | 24 (25.53) | 6 (27.27) |  |
| Histological grade |  |  |  |  |  |  |
| High | 23 (24.47) | 7 (31.82) | 0.7729 | 25 (26.60) | 5 (22.73) | 0.8405 |
| Moderate | 20 (21.28) | 4 (18.18) |  | 20 (21.28) | 4 (18.18) |  |
| Poor | 51 (54.26) | 11 (50.00) |  | 49 (52.13) | 13 (59.09) |  |
| Tumor associated immune cells |  |  |  |  |  |  |
| Low | 53 (56.38) | 11 (50.00) | 0.7613 | 57 (60.64) | 7 (31.82) | 0.0272 |
| High | 41 (43.62) | 11 (50.00) |  | 37 (39.36) | 15 (68.18) |  |
| SerumCA125 |  |  |  |  |  |  |
| High | 19 (26.03) | 4 (22.22) | 0.9761 | 20 (27.40) | 3 (16.67) | 0.5251 |
| Low | 54 (73.97) | 14 (77.78) |  | 53 (72.60) | 15 (83.33) |  |
| SerumCEA |  |  |  |  |  |  |
| High | 27 (34.18) | 6 (31.58) | 1 | 28 (35.00) | 5 (27.78) | 0.7567 |
| Low | 52 (65.82) | 13 (68.42) |  | 52 (65.00) | 13 (72.22) |  |
| SerumCA199 |  |  |  |  |  |  |
| High | 67 (78.82) | 14 (70.00) | 0.5826 | 64 (75.29) | 17 (85.00) | 0.526 |
| Low | 18 (21.18) | 6 (30.00) |  | 21 (24.71) | 3 (15.00) |  |

Continued Table S5 Associations between the locations of CD68+ immune cells and clinicopathological characteristics in pancreatic ductal adenocarcinoma

| characteristics | CD68 TC | | *p*-value | CD68 IMs | | *p*-value |
| --- | --- | --- | --- | --- | --- | --- |
|  | Low(n=94) | High(n=22) |  | Low(n=94) | High(n=22) |  |
| Nerve Invasion |  |  |  |  |  |  |
| Absence | 28 (29.79) | 5 (22.73) | 0.6905 | 26 (27.66) | 7 (31.82) | 0.8992 |
| Presence | 66 (70.21) | 17 (77.27) |  | 68 (72.34) | 15 (68.18) |  |
| Extra-pancreatic Invasion |  |  |  |  |  |  |
| Absence | 14 (14.89) | 3 (13.64) | 1 | 15 (15.96) | 2 (9.09) | 0.6277 |
| Presence | 80 (85.11) | 19 (86.36) |  | 79 (84.04) | 20 (90.91) |  |
| Tumor-stroma ratio |  |  |  |  |  |  |
| Stromal low | 66 (70.21) | 16 (72.73) | 1 | 62 (65.96) | 20 (90.91) | 0.0399 |
| Sromal high | 28 (29.79) | 6 (27.27) |  | 32 (34.04) | 2 (9.09) |  |
| Stroma maturity |  |  |  |  |  |  |
| Mature | 26 (27.66) | 5 (22.73) | 0.5897 | 28 (29.79) | 3 (13.64) | 0.3039 |
| Moderate | 24 (25.53) | 8 (36.36) |  | 25 (26.60) | 7 (31.82) |  |
| Immature | 44 (46.81) | 9 (40.91) |  | 41 (43.62) | 12 (54.55) |  |

Table S6 Associations between the locations of CD206+ immune cells and clinicopathological characteristics in pancreatic ductal adenocarcinoma

| characteristics | CD206 TC | | *p*-value | CD206 IMs | | *p*-value |
| --- | --- | --- | --- | --- | --- | --- |
|  | Low(n=100) | High(n=16) |  | Low(n=94) | High(n=22) |  |
| Age |  |  |  |  |  |  |
| <60 years | 49 (49.00) | 6 (37.50) | 0.5581 | 46 (48.94) | 9 (40.91) | 0.6588 |
| ≥60 years | 51 (51.00) | 10 (62.50) |  | 48 (51.06) | 13 (59.09) |  |
| Sex |  |  |  |  |  |  |
| Male | 58 (58.00) | 12 (75.00) | 0.3099 | 58 (61.70) | 12 (54.55) | 0.7072 |
| Female | 42 (42.00) | 4 (25.00) |  | 36 (38.30) | 10 (45.45) |  |
| pT stage |  |  |  |  |  |  |
| T1 | 18 (18.00) | 1 (6.25) | 0.2186 | 17 (18.09) | 2 (9.09) | 0.5595 |
| T2 | 57 (57.00) | 8 (50.00) |  | 51 (54.26) | 14 (63.64) |  |
| T3 | 25 (25.00) | 7 (43.75) |  | 26 (27.66) | 6 (27.27) |  |
| pN stage |  |  |  |  |  |  |
| N0 | 47 (47.00) | 8 (50.00) | 0.4895 | 48 (51.06) | 7 (31.82) | 0.0946 |
| N1 | 43 (43.00) | 5 (31.25) |  | 38 (40.43) | 10 (45.45) |  |
| N2 | 10 (10.00) | 3 (18.75) |  | 8 (8.51) | 5 (22.73) |  |
| M stage |  |  |  |  |  |  |
| M0 | 96 (96.00) | 12 (75.00) | 0.0109 | 90 (95.74) | 18 (81.82) | 0.0639 |
| M1 | 4 (4.00) | 4 (25.00) |  | 4 (4.26) | 4 (18.18) |  |
| TNM grade |  |  |  |  |  |  |
| I+II | 77 (77.00) | 9 (56.25) | 0.1464 | 72 (76.60) | 14 (63.64) | 0.3275 |
| III+IV | 23 (23.00) | 7 (43.75) |  | 22 (23.40) | 8 (36.36) |  |
| Histological grade |  |  |  |  |  |  |
| High | 24 (24.00) | 6 (37.50) | 0.3639 | 24 (25.53) | 6 (27.27) | 0.9344 |
| Moderate | 20 (20.00) | 4 (25.00) |  | 19 (20.21) | 5 (22.73) |  |
| Poor | 56 (56.00) | 6 (37.50) |  | 51 (54.26) | 11 (50.00) |  |
| Tumor associated immune cells |  |  |  |  |  |  |
| Low | 59 (59.00) | 5 (31.25) | 0.0716 | 55 (58.51) | 9 (40.91) | 0.209 |
| High | 41 (41.00) | 11 (68.75) |  | 39 (41.49) | 13 (59.09) |  |
| SerumCA125 |  |  |  |  |  |  |
| High | 19 (24.68) | 4 (28.57) | 1 | 19 (26.39) | 4 (21.05) | 0.8577 |
| Low | 58 (75.32) | 10 (71.43) |  | 53 (73.61) | 15 (78.95) |  |
| SerumCEA |  |  |  |  |  |  |
| High | 27 (31.76) | 6 (46.15) | 0.4794 | 28 (35.90) | 5 (25.00) | 0.5126 |
| Low | 58 (68.24) | 7 (53.85) |  | 50 (64.10) | 15 (75.00) |  |
| SerumCA199 |  |  |  |  |  |  |
| High | 70 (77.78) | 11 (73.33) | 0.9622 | 65 (76.47) | 16 (80.00) | 0.9663 |
| Low | 20 (22.22) | 4 (26.67) |  | 20 (23.53) | 4 (20.00) |  |

Continued Table S6 Associations between the locations of CD206+ immune cells and clinicopathological characteristics in pancreatic ductal adenocarcinoma

| characteristics | CD206 TC | | | *p*-value | CD206 IMs | | | *p*-value |
| --- | --- | --- | --- | --- | --- | --- | --- | --- |
|  | Low(n=100) | | High(n=16) |  | Low(n=94) | | High(n=22) |  |
| Nerve Invasion |  |  | |  |  |  | |  |
| Absence | 28 (28.00) | 5 (31.25) | | 1 | 29 (30.85) | 4 (18.18) | | 0.3559 |
| Presence | 72 (72.00) | 11 (68.75) | |  | 65 (69.15) | 18 (81.82) | |  |
| Extra-pancreatic Invasion |  |  | |  |  |  | |  |
| Absence | 15 (15.00) | 2 (12.50) | | 1 | 13 (13.83) | 4 (18.18) | | 0.8534 |
| Presence | 85 (85.00) | 14 (87.50) | |  | 81 (86.17) | 18 (81.82) | |  |
| Tumor-stroma ratio |  |  | |  |  |  | |  |
| Stromal low | 70 (70.00) | 12 (75.00) | | 0.9107 | 66 (70.21) | 16 (72.73) | | 1 |
| Sromal high | 30 (30.00) | 4 (25.00) | |  | 28 (29.79) | 6 (27.27) | |  |
| Stroma maturity |  |  | |  |  |  | |  |
| Mature | 29 (29.00) | 2 (12.50) | | 0.2728 | 25 (26.60) | 6 (27.27) | | 0.8412 |
| Moderate | 28 (28.00) | 4 (25.00) | |  | 27 (28.72) | 5 (22.73) | |  |
| Immature | 43 (43.00) | 10 (62.50) | |  | 42 (44.68) | 11 (50.00) | |  |


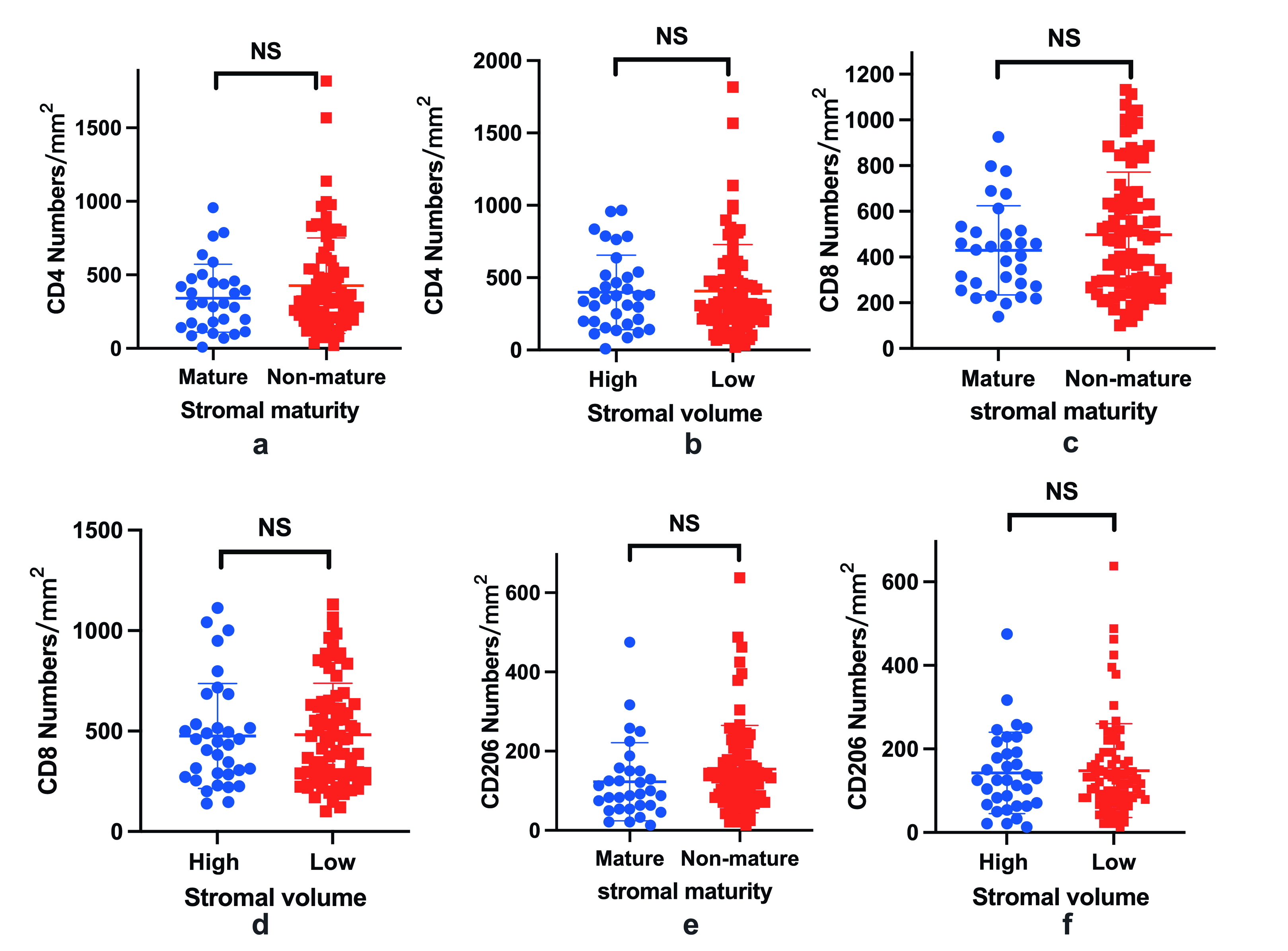


Figure S1. The relationships of stromal volume and maturity with CD4，CD8 and CD206 positive cells in whole slides. (a) The relationships between stromal maturity and CD4+ cells. (b) The relationships between stromal volume and CD4+ cells. (c) The relationships between stromal maturity and CD8+ cells. (d) The relationships between stromal volume and CD8+ cells. (e) The relationships between stromal maturity and CD206+ cells. (f) The relationships between stromal volume and CD206+ cells. Mann-Whitney U test, NS: no statistic significance.


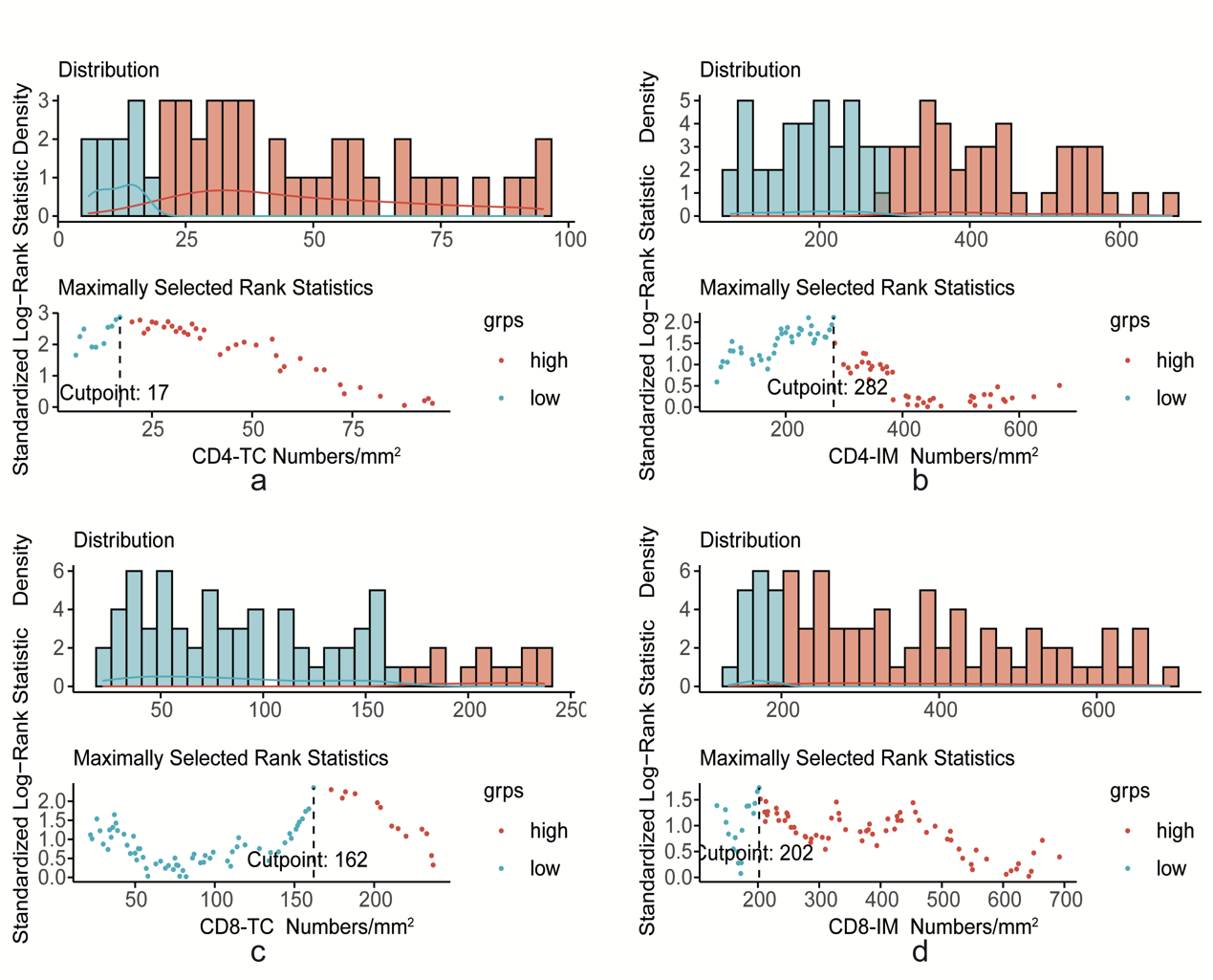


Figure S2. The distribution of infiltrating immune cells (CD4&CD8) and the chosen of most optminal cut-off value. (a) The distribution of CD4+ immune cells at TC does not conform to the normal distribution. The most optminal cut-off value is 17 cells/mm^2^. (b) The distribution of CD4+ immune cells at IMs does not conform to the normal distribution. The most optminal cut-off value is 282 cells/mm^2^. (c) The distribution of CD8+ immune cells at TC does not conform to the normal distribution. The most optminal cut-off value is 162 cells/mm^2^. (d) The distribution of CD8+ immune cells at IMs does not conform to the normal distribution. The most optminal cut-off value is 202 cells/mm^2^.


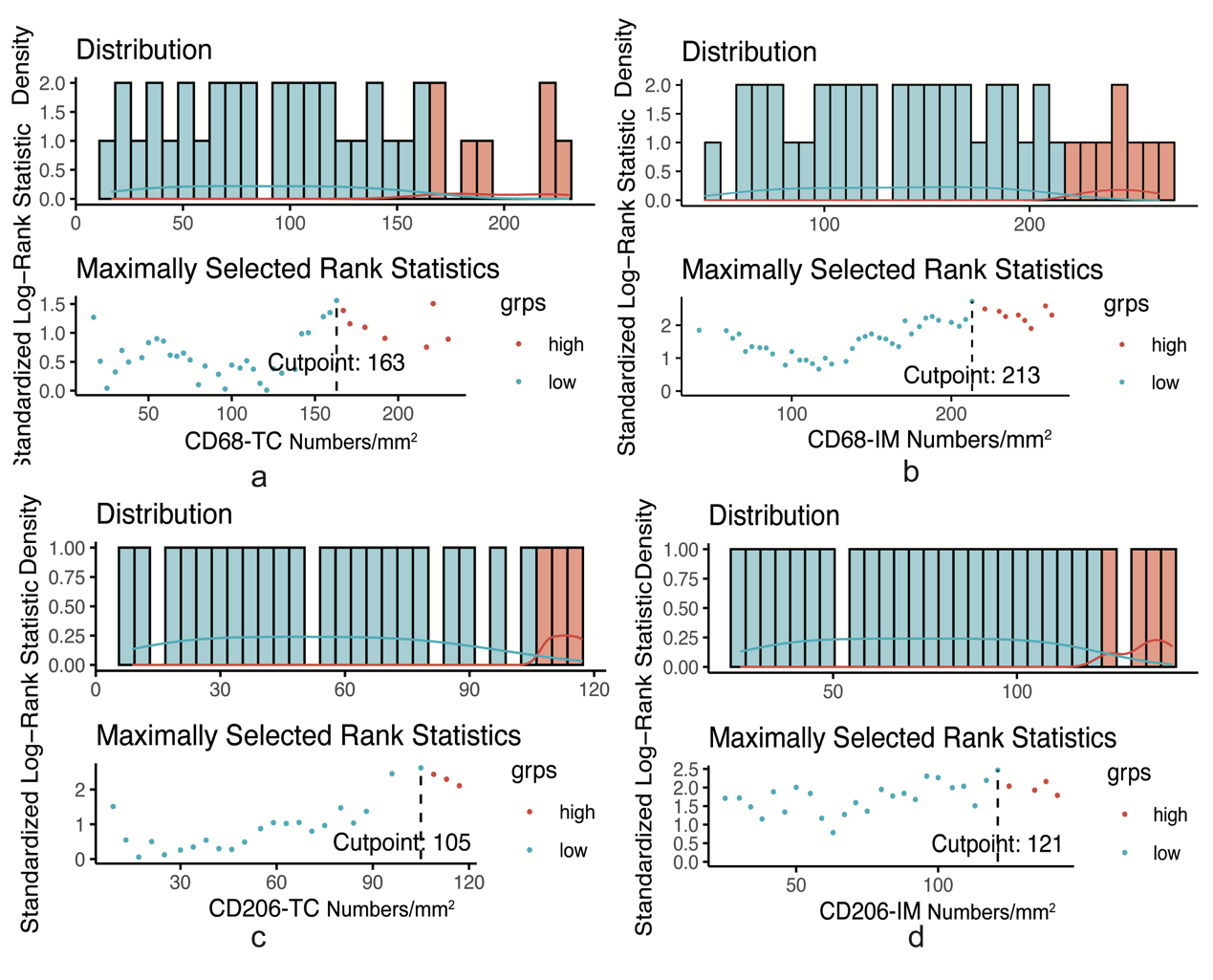


Figure S3. The distribution of infiltrating immune cells (CD68&CD206)and the chosen of most optminal cut-off value.(a) The distribution of CD68+ immune cells at TC does not conform to the normal distribution. The most optminal cut-off value is 163 cells/mm^2^. (b) The distribution of CD68+ immune cells at IMs does not conform to the normal distribution. The most optminal cut-off value is 213 cells/mm^2^. (c) The distribution of CD206+ immune cells at TC does not conform to the normal distribution. The most optminal cut-off value is 10 cells/mm^2^. (d) The distribution of CD206+ immune cells at IMs does not conform to the normal distribution. The most optminal cut-off value is 121 cells/mm^2^.


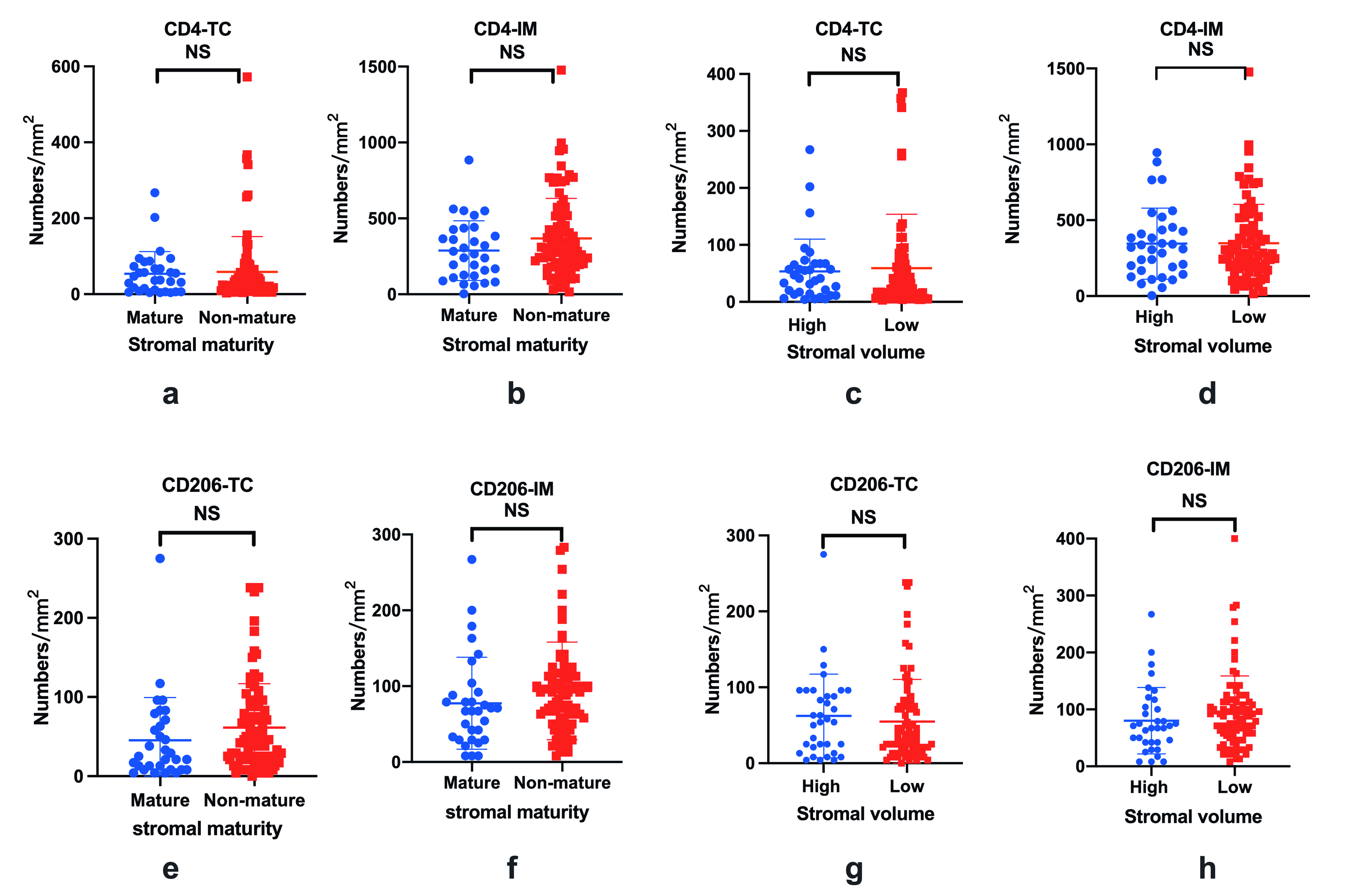


Figure S4. Relationships of tumor stromal maturity and volume with different locations of CD4 and CD206 positive cells in pancreatic ductal adenocarcinoma. (a) Relationships of stromal maturity and CD4+ cells in tumor center,(b) in invasive margin.(c) Relationships of stromal volume and CD4+ cells in tumor center,(d) in invasive margin. (e)Relationships of stromal maturity and CD206+ cells in tumor center,(f) in invasive margin.(g) Relationships of stromal volume and CD206+ cells in tumor center,(h) in invasive margin. Mann-Whitney U test, NS: no statistic significance.


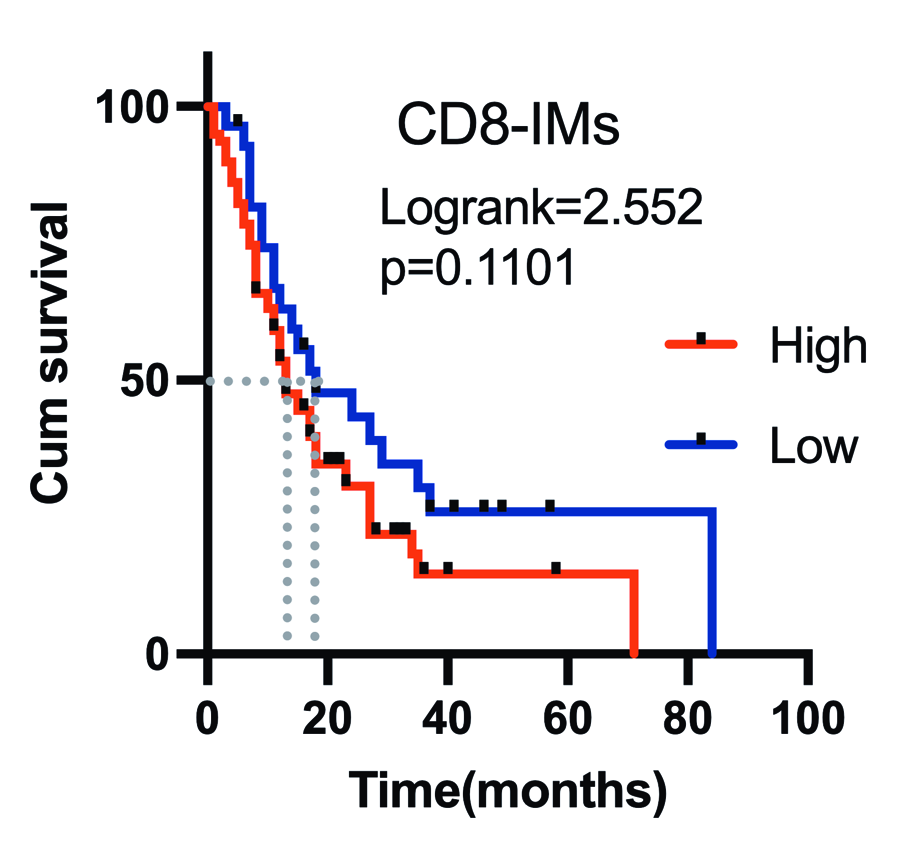


Figure S5. Kaplan Meier survival analysis for patients with different quantities of CD8+immune cells in areas of invasive margin.
